# Revealing hidden biodiversity: Novel insights on reptile and amphibian distribution in western Ecuador

**DOI:** 10.1002/ece3.11401

**Published:** 2024-06-06

**Authors:** Keyko Cruz‐García, Natalia Zapata‐Salvatierra, Juan C. Sánchez‐Nivicela, Nadia Chauca, Sascha Matecki, Julian Perez‐Correa

**Affiliations:** ^1^ Instituto de Biodiversidad Tropical IBIOTROP, Laboratorio de Zoología Terrestre, Museo de Zoología Universidad San Francisco de Quito USFQ Quito Ecuador; ^2^ Museo de Zoología Universidad Técnica Particular de Loja Loja Ecuador; ^3^ Facultad de Ciencias Naturales Universidad de Guayaquil Guayaquil Ecuador; ^4^ Laboratorio de Zoología, Facultad de Ciencias de la Vida Escuela Superior Politécnica del Litoral, ESPOL Guayaquil Ecuador; ^5^ Grupo de Investigación, Evolución y Ecología de Fauna Neotropical, Facultad de Ciencias Universidad Nacional de Colombia Bogotá D.C. Colombia; ^6^ Instituto Nacional de Biodiversidad Quito Ecuador; ^7^ Fundación para la Conservación e Investigación JaPu Guayaquil Ecuador

**Keywords:** *Agalychnis*, Cañar, Guayas, *Hyloscirtus*, *Imantodes*, new records, *Pristimantis*, *Urotheca*

## Abstract

We present notable distributional updates for 14 species from western Ecuador (seven amphibians and seven reptiles). Our findings include the northernmost confirmed sighting of *Pristimantis kuri* and the southernmost documented record of *Imantodes inornatus* and *Lepidoblepharis buchwaldi*. Additionally, we document new records and notes on the distribution range of *Agalychnis spurrelli*, *Hyloscirtus alytolylax*, *Engystomops montubio*, *Pristimantis muricatus*, *Pristimantis nyctophylax*, *Pristimantis walkeri*, *Chironius flavopictus*, *Chironius grandisquamis*, *Dendrophidion graciliverpa*, *Ninia schmidti*, and *Urotheca fulviceps*. These observations significantly contribute to filling information gaps in our understanding of these species' distributions. The data, derived from samples collected across diverse forested areas in the western region of Ecuador (provinces of Bolívar, Cañar, Guayas, El Oro, and Los Ríos), provide valuable insights into the ecology and conservation of these species.

## Graphical abstract

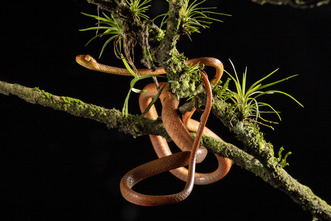

## Resumen

Presentamos importantes actualizaciones distribucionales para 14 especies del oeste de Ecuador (siete anfibios y siete reptiles). Nuestros hallazgos incluyen el avistamiento confirmado más al norte de *Pristimantis kuri* y el registro documentado más al sur de *Imantodes inornatus* y *Lepidoblepharis buchwaldi*. Además, documentamos nuevos registros y notas sobre el rango de distribución *de Agalychnis spurrelli*, *Hyloscirtus alytolylax*, *Engystomops montubio*, *Pristimantis muricatus*, *Pristimantis nyctophylax*, *Pristimantis walkeri*, *Chironius flavopictus*, *Chironius grandisquamis*, *Dendrophidion graciliverpa*, *Ninia schmidti* y *Urotheca fulviceps*. Estas observaciones contribuyen significativamente a llenar vacíos de información en nuestra comprensión de la distribución de estas especies. Los datos, derivados de muestras recolectadas en diversas áreas forestales de la región occidental de Ecuador (provincias de Bolívar, Cañar, Guayas, El Oro y Los Ríos), proporcionan valiosos conocimientos sobre la ecología y conservación de estas especies.

## 1 INTRODUCTION

Ecuador is an outstanding megadiverse country, with ecosystems and species found nowhere else on the planet. Nonetheless, the country is grappling with a persistent loss of habitats that threatens species with extinction (Cuesta et al., 2017), primarily due to land‐use changes and climate variations (Griffis‐Kyle et al., 2018). Cuesta et al. (2017) emphasize that 47% of coastal habitats in Ecuador are experiencing continuous deforestation, a trend often linked to the expansion of permanent crops, notably evident in the Guayas River basin (Sierra, 2013). Conversely, the province of Los Ríos emerges as one of the most significantly impacted regions in terms of biodiversity due to the extensive habitat destruction driven by agricultural expansion (Cornejo, 2011). It is therefore evident that anthropogenic activities play a pivotal role in altering the distribution patterns of species (Turvey et al., 2018).

Home to a remarkable diversity of amphibians and reptiles, Ecuador benefits from its location within critical biodiversity hotspots: the Tumbes‐Chocó‐Magdalena and Tropical Andes regions. This positioning makes it a pivotal region for conservation efforts in South America, as it fosters elevated levels of diversity and endemism (Myers et al.,&amp;#x000A0;^2000^). However, this great herpetofaunal diversity faces severe pressure from anthropogenic activities, rendering them one of the most threatened vertebrate groups (IUCN SSC Amphibian Specialist Group, 2023). Amphibians and reptiles play crucial roles as indicators of ecosystem health, as they are highly sensitive to environmental stressors (Prasad et al., 2018; Steinke, 2016). For example, climate change represents a critical challenge for amphibians as it induces significant alterations in their metabolic processes, body growth, development, and reproductive patterns. Moreover, it notably increases their susceptibility to diseases (Cheza et al., 2020; Griffis‐Kyle et al., 2018). In response to these threats, they often resort to strategies such as migration (Sinsch, 1990). However, several types of herpetofauna found in lowland areas struggle to adapt to elevated temperatures. With ongoing and unchecked global warming, the likelihood of extinction rises for various taxa, including herpetofauna, plants, and invertebrates. Additionally, a significant number of these species likely face challenges in moving or migrating to different locations due to their limited mobility (Laurance et al., 2011). Thus, studies have focused on assessing responses to environmental changes by examining shifts in distribution ranges (Ehrlén & Morris, 2015).

Despite the adversities confronting these species, the research and published studies on the composition of herpetofaunal communities in the central and southwestern ecosystems of Ecuador are scarce. The continual and rapid loss of habitats exacerbates the lack of information on amphibians and reptiles (Armijos‐Ojeda et al., 2021; Kleemann et al., 2022). Consequently, some species, such as *Atractus microrhynchus*, *Chironius flavopictus*, or *Pristimantis tenebrionis*, have not been reported since their initial description. Nevertheless, there is a gradual emergence of studies shedding light on novel perspectives regarding the distribution of herpetofauna in Ecuador (Amador, Ayala‐Varela, et al., 2017; Amador, Gómez, et al., 2017; Arteaga et al., 2022; Arteaga & Harris, 2023; Ayala‐Varela et al., 2015; Brito‐Zapata et al., 2021; Carvajal‐Endara et al., 2019; Cruz et al., 2017; Cruz‐García et al., 2020, 2023; Culebras et al., 2020; Parra et al., 2020; Passos et al., 2012; Reyes‐Puig et al., 2019; Székely et al., 2021; Torres‐Carvajal et al., 2017; Yánez‐Muñoz et al., 2016; Yánez‐Muñoz et al., 2021).

Information about the distribution of the species is crucial for comprehensively understanding and documenting species composition in remote locations, such as the southwest western foothills of the Andes in Ecuador. This manuscript reports on several new extensions of distribution ranges observed in the provinces of Bolivar, Los Ríos, Guayas, El Oro, and Cañar, which are adjacent in the southwest region of Ecuador. Los Ríos, Guayas, and most of El Oro represent lowland areas, while Cañar, Bolivar, and some areas of El Oro encompass highland regions. These study sites are some of the last remnants of tropical forest remaining in the provinces of Bolivar, Cañar, Guayas, El Oro, and Los Ríos and are currently threatened by agricultural expansion, logging, mining, and poaching (Sierra et al., 2021; Torres et al., 2020).

## 2 METHODS

The data were collected during scientific exploratory expeditions in 10 tropical forests in western Ecuador, from January 2014 to December 2023, utilizing non‐systematic sampling, a search that was both free and unrestricted, visual recordings, and manual capture methods (Rödel & Ernst, 2004). According to the Ministerio del Ambiente del Ecuador (2013), 4 of the 10 forests sampled presented a seasonal foothill evergreen forest of the western Los Andes Mountain Range; these sites were as follows: (1) the Cerro de Hayas Provincial Natural Recreation Area (camp point: 2°43′50.9″ S, 79°37′43″ W, alt. 120 m) in the foothills of the Molleturo‐Mollepungo mountain range, Naranjal canton, Guayas Province, Ecuador. Cerro de Hayas is a private reserve that protects 631 ha of forests in mountainous landscapes with streams and waterfalls. (2) Rancho Alemán (camp point: 2°20′13.6″ S, 79°12′46.8″ W, alt. 235 m) in the western foothills of Los Andes Mountain Range, El Triunfo town, Guayas Province, Ecuador. Rancho Alemán is an ecotourism farm that has 25 ha of forest in mountainous landscapes with streams and is surrounded by the Blanco River. (3) San Pablo town (camp point: 2°20′55″ S, 79°10′28.9″ W, alt. 431 m) in the western foothills of Los Andes Mountain Range, Cañar canton, Cañar Province, Ecuador. A small town surrounded by mountains, ravines, and small waterfalls, where there are patches of forests and areas dedicated to agriculture. (4) Cascadas de Manuel (camping point: 3°12′23.1″ S, 79°44′9.8″ W, alt. 182 m) located in the El Guabo town, El Oro Province. It has mature forest extension that occupies almost the entire micro‐basin with a dense tree cover, with an interior vegetation based on herbaceous and shrubs. Three forests are evergreen foothill forests of the western Los Andes Mountain Range, which were as follows: (5) the surroundings of the El Progreso farm (camping point: 2°7′39.4″ S, 79°7′17″ W, alt. 944 m) in the western foothills of Los Andes Mountain Range, Chillanes town, Bolivar Province, Ecuador. This site is a tourist farm that presents forest patches with mountainous landscapes, streams, ravines, waterfalls, and crop areas. (6) El bosque del Amigo farm (camping point: 2°17′3.5″ S, 79°6′23″ W, alt. 879 m) in the western foothills of Los Andes Mountain Range, Cañar town, Cañar Province, Ecuador. This site is an ecotourism farm that has 300 ha, large tracts of forest with mountains, a large number of small streams, and small areas for agriculture. (7) The surroundings of Ocaña (camping point: 2°29′3.7″ S, 79°15′12.2″ W, alt. 946 m) in the western foothills of Los Andes Mountain Range, Cañar town, Cañar province, Ecuador. There were large patches of forest on sloping slopes of the Cañar River. The forests were dense with predominantly shrubby vegetation and herbaceous in its interior.

Other ecosystems were evergreen seasonal lowland forest of Jama‐Zapotillo that was (8) Pedro Franco Dávila Protected Forest (camp point: 1°14′44.2″ S, 79°39′36″ W, alt. 50 m) in the central region of the Ecuadorian coast, Palenque town, Los Ríos Province, Ecuador. This site has 140 ha of protected forest, a tropical humid forest with two climatic seasons: rainy season (January–May) and dry season (June–December). In addition, it presents mainland forests, rivers, streams, and small swampy areas. (9) The surroundings of Mocache town (camping point: 1°9′40.9″ S, 79°30′29.4″ W, alt. 57 m), Los Ríos Province, Ecuador. It is located on the banks of the Vinces River and has small fragments of intervened forest. Its vegetation is sparse, it has few shrubs and abundant leaf litter. (10) The surroundings of Macul (camping point: 1°36′22.1″ S, 79°50′58.8″ W, alt. 18 m), Vinces canton, Los Ríos Province, Ecuador. Located next to the river, it has small remnants of intervened forest rich in litter, but with very little shrub and herbaceous vegetation.

Specimens were hand‐captured, photographed, and euthanized using 2% roxicaine anesthetic solution, fixed in 10% formalin, and preserved in 70% ethanol. Before fixation, tissue samples were taken from the collected individuals and preserved in 96% ethanol, following the method described by Székely et al. (2016). The specimens are deposited in the following museums: Museo de Zoología de la Universidad Técnica Particular de Loja (MUTPL), Loja, Ecuador; Museo de Zoología de la Universidad del Azuay (MZUA), Cuenca, Ecuador; Museo de Zoología de la Pontificia Universidad Católica del Ecuador (QCAZ), Quito, Ecuador; and Museo de Zoología, Universidad San Francisco de Quito (ZSFQ), Quito, Ecuador. Snout–vent length (SVL) measurements were taken with digital calipers and rounded to the nearest 0.1 mm for small specimens and with a tape measure for longer specimens. The coordinates and elevation were taken with Garmin GPSMAP 62st GPS equipment. Specimens were collected under permit No. MAE‐DPALR‐UPN‐UB‐2015‐002, 008‐2015‐IC‐FLO/FAU‐DPG/MAE, MAAE‐DBI‐DBI‐CM‐2022‐0222, MAAE‐ARSFC‐2022‐2204, and MAATE‐ARSFC‐2023‐0063 approved by the Ministerio del Ambiente, Agua y Transición Ecológica.

## 3 RESULTS

We present new records of seven amphibians and seven reptiles in western Ecuador corresponding to the provinces of Bolivar, Cañar, Guayas, El Oro, and Los Rios. In the Guayas Province, this study reports the first records of *Imantodes inornatus*, *Pristimantis kuri*, *Pristimantis nyctophylax*, and *Urotheca fulviceps*. It should be noted that the record of *Chironius flavopictus* in Guayas has been updated, with no records collected since its initial discovery by Werner (1909). Likewise, the recently validated species *Ninia schmidti*, which according to Arteaga and Harris (2023) was collected between 1901 and 1902 in Guayaquil, Guayas, Ecuador. This study also presents the northernmost reported record of *Pristimantis kuri* (first record in the province of Bolívar) and the southernmost known records of *Imantodes inornatus* and *Lepidoblepharis buchwaldi*. Additionally, new records for the Cañar Province include *Agalychnis spurrelli*, *Dendrophidion graciliverpa*, *Lepidoblepharis buchwaldi*, *Pristimantis kuri*, *Pristimantis muricatus*, *Pristimantis nyctophylax*, *Pristimantis walkeri*, and *Urotheca fulviceps*. And finally, the first record of *Engystomops montubio* for the province of Los Ríos further enriched our knowledge about the distribution of this species.

### Class amphibia: Order Anura: Family Hylidae

### *Agalychnis spurrelli* Boulenger, 1913

**New Records**. ECUADOR—**Cañar** • Cañar Canton, San Pablo town; 2°20′26.9″ S, 79°10′42.2″ W; alt. 420 m; 12.III.2023; Keyko Cruz‐García leg; found perched on a branch between 120 and 190 cm above a water puddle next to a secondary road; SVL 64 mm; SVL 62 mm; 2 ♂; ZSFQ5182 and ZSFQ5183.

The individuals of this species were exclusively found along the edge of a secondary road near a water puddle, and they were not present within lightly intervened forest areas.

**FIGURE 1:**
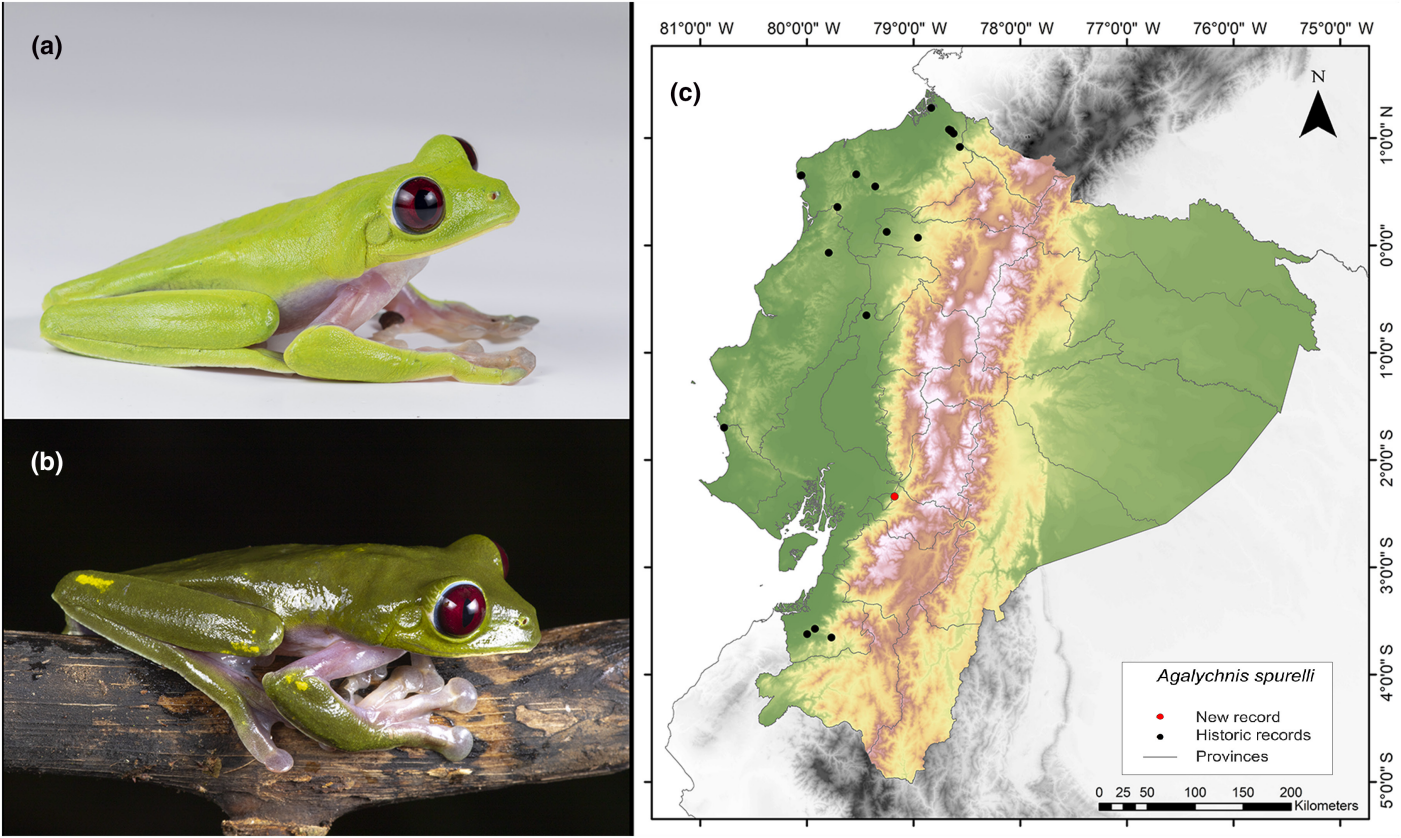
Photographs of *Agalychnis spurrelli* (a and b) and its distribution map (c) depicting historic records in black dots and the new record in red dots. The images belong to the specimen registered in this study.

**Identification.** (Figure 1a,b) Medium‐ to large‐sized frog with extensive interdigital membranes; yellow, orange, pale pink, or pale purple color on flanks and limbs; lacking dark vertical stripes; usually presents cream‐colored warts bordered in black on the dorsal side; smooth dorsal skin; granular ventral surface; indistinct parotoid glands; visible tympanum; acuminated or subacuminated snout in dorsal view; round and conspicuous rostral ridge; concave loreal region; thin, moderately flared lips; protruding, large eyes; dark crimson red iris with thin black reticulations; vertical elliptical pupil; nictitating membrane with golden reticulations; spiny nuptial pad at the base of the thumb in adult males; finger I (manual) and toe I (pedial) shorter than finger II and toe II, respectively; and large disks, all larger than the tympanum (Ortega‐Andrade, 2008; Ron et al., 2022; Savage, 2002).

**Distribution.** It is distributed from the central western lowlands of Costa Rica to the Pacific lowlands of Colombia (Valle del Cauca and Chocó) and Ecuador. According to Ortega‐Andrade (2008), its distribution in Ecuador covers an area of 19,550 km^2^ and is present in seven provinces of Ecuador (Esmeraldas, Los Ríos, Manabí, Pichincha, Carchi, Santa Elena, El Oro). Its altitudinal range extends from 70 to 1000 m.a.s.l. (Ortega‐Andrade, 2008; Ron et al., 2022; Yánez‐Muñoz et al., 2019). The current record fills a 328 km information gap in the known distribution of the western foothills of the Andes (Figure 1c). The northernmost nearby locality is positioned 189 km away (Hacienda Cerro Chico, Los Ríos Province; Ortega‐Andrade, 2008), and the southernmost is located at a distance of 159 km (El Remolino, El Oro Province; Pontificia Universidad Católica del Ecuador 2021a).

### *Hyloscirtus alytolylax* Duellman, 1972

**New Records**. ECUADOR—**Cañar** • Cañar Canton, El bosque del Amigo farm; 2°16′34.6″ S, 79°5′55″ W; alt. 668 m; 17.IX.2023; Keyko Cruz‐García leg; found perched on a leaf 3 m high over a ravine in secondary forest on one bank and a small banana plantation on the other; SVL 37,92 mm; 1 (sex indet.); MUTPL‐A 1671. • Cañar Canton, San Antonio de Paguancay parish, Ocaña; 2°29′56.7″ S, 79°14′52.4″ W; alt. 495 m; 6.VII.2008, 24.II.2014,23.IX.2015; Juan C. Sánchez‐Nivicela, Verónica L. Urgiles, Elvis Celi leg. In the stream edge; SVL 18.78 mm; 18.86 mm; 18.70 mm; 3 ♂; MZUA‐An.0043; MZUA‐An.0982; and MZUA‐An.1536.

**FIGURE 2:**
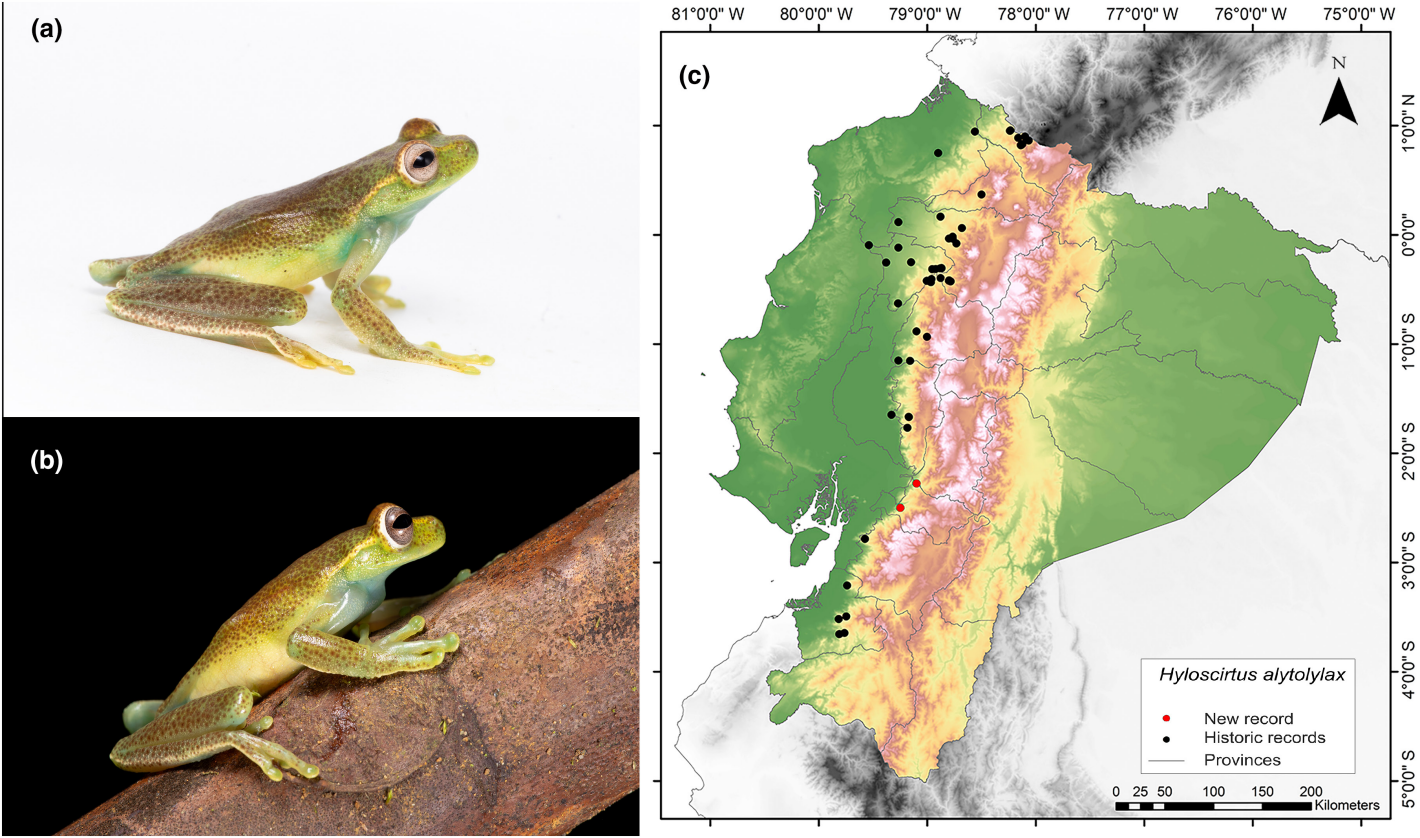
Photographs of *Hyloscirtus alytolylax* (a and b) and its distribution map (c) depicting historic records in black dots and the new record in red dots. The images belong to the specimen registered in this study.

**Identification.** (Figure 2a,b) It is a medium‐sized frog that has the characteristics of having a head slightly wider than the body; top of head flat, snout short, rounded in dorsal view, rounded in lateral view in males and truncated in females; rostral edge rounded; concave loreal region; nostrils not protruding; internal area slightly depressed; thin, round lips; eyes not very protruding; weak supratympanic fold, curved downwards, obscuring the upper edge of the eardrum; tympanic ring evident ventrally; eardrum slightly smaller than half the diameter of the eye; axillary membrane absent; forearm robust, with ulnar fold; short fingers with short discs; disc of finger III slightly wider than eardrum; rudimentary membranes between the fingers; hind limbs moderately robust; dermal fold in the knee; calcar usually absent; internal tarsal fold absent; external tarsal fold extends along the entire length of the tarsus; long toes; discs slightly smaller than those of the fingers; extensive webbing between toes; cloacal opening directed posteriorly at the upper level of the thighs; belly skin weakly granular; skin on other body surfaces smooth; and bilobed and subgular vocal sac. A cream line extends along the rostral canthus, eyelid margin, and supratympanic fold (Duellman, 1972).

**Distribution.** This species is known to occur on the Pacific slopes of the Andes in southern Colombia and through western Ecuador to Oro Province, between 400 and 2000 m.a.s.l. (Frost, 2023; Guayasamin et al., 2015). The current records enhance our understanding of the species distribution in the western foothills of the Andes, filling an information gap of 120 km (Figure 2c). The closest northern locality is positioned at a distance of 57 km (Balzapamba, Bolívar Province; GBIF, 2024), while the closest southern one is situated 48 km away (San Miguel, Guayas Province; Instituto Nacional de Biodiversidad, 2022).

### Class amphibia: Order Anura: Family Leptodactylidae

### *Engystomops montubio* (Ron, Cannatella, and Coloma, 2004)

**New Records**. ECUADOR—**Los Ríos** • Palenque Canton, Pedro Franco Dávila Protected Forest; 1°14′25.8″ S, 79°39′59.4″ W; alt. 72 m; 12.III.2016; Keyko Cruz‐García leg; found foraging in the leaf litter next to a trail.; SVL 22,71 mm; 1 ♀; MZUA‐An.1664. • Mocache Canton; 1°9′40.7″ S, 79°30′29.2″ W; alt. 57 m; 12.I.2014; Juan C. Sánchez‐Nivicela, Verónica L. Urgiles, Elvis Celi leg; between semi‐submerged leaf litter in a small puddle near a wetland; SVL 18.78 mm; 18.86 mm; 2 ♂; MZUA‐An.0919; and MZUA‐An.0920.

**FIGURE 3:**
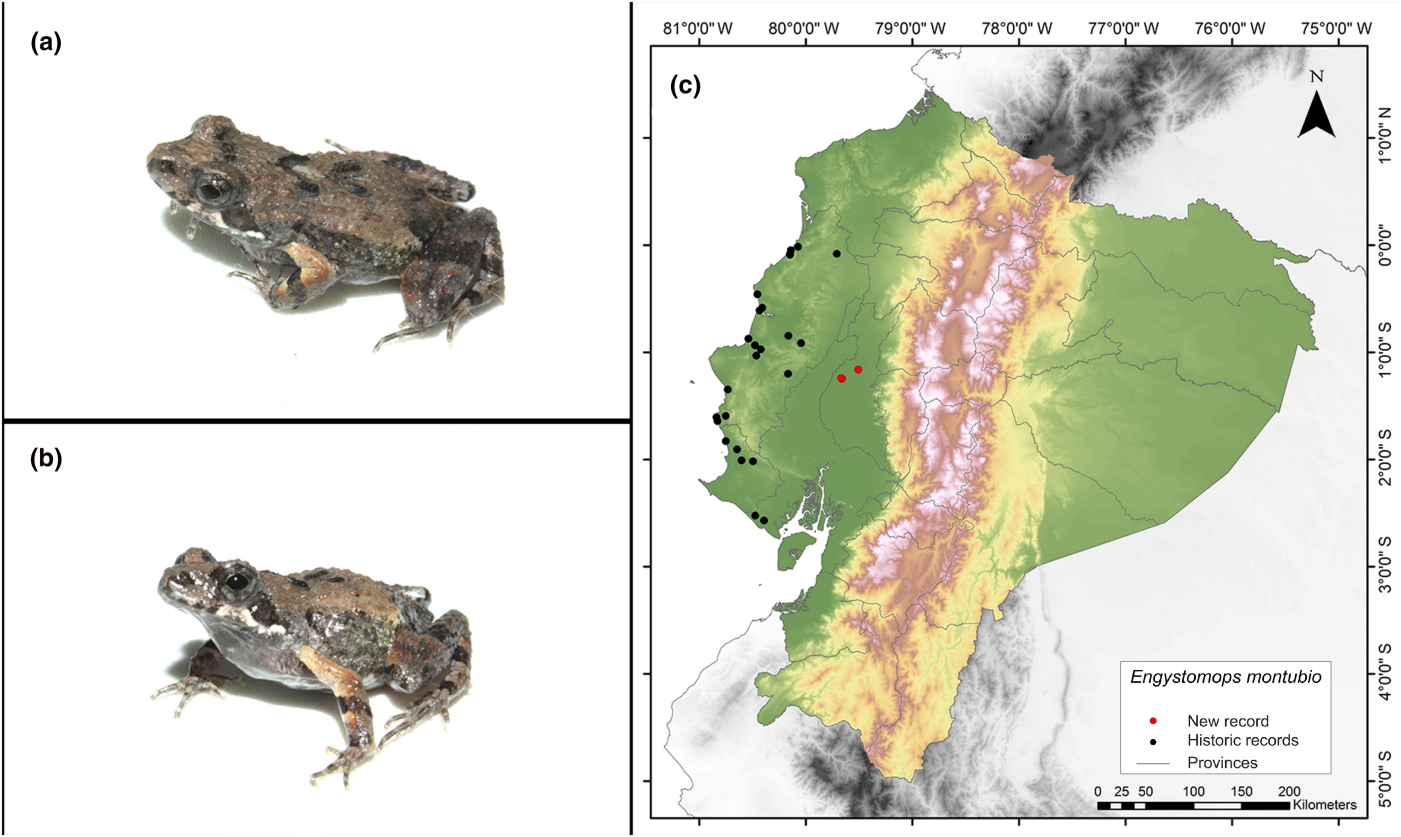
Photographs of *Engystomops montubio* (a and b) and its distribution map (c) depicting historic records in black dots and the new record in red dots. The images belong to the specimen registered in this study.

**Identification.** (Figure 3a,b) It is a very small frog, light brown in color with dark spots, and a whitish belly speckled with brown; it has an average SVL (snout–vent length) in males of 20.6 mm and females of 18.52 mm; subacuminate snout in dorsal view and rounded in lateral view; evident tympanic ring, dorsally hidden; non‐tuberculated tympanic membrane; finger I shorter than finger II; nuptial pads present; absent tarsal tubercle; the dorsum bears numerous rounded or subconical tubercles; glands present on the flank; parotoid glands present; maxillae and premaxillae present; vomerine odontophore absent; and vomerine dentigerous process thin and pointed (like a spine) (Ron et al., 2004).

**Distribution.** This frog is endemic to western Ecuador and is distributed in the provinces of Manabí, Santa Elena, and Guayas. Its altitudinal range extends from sea level of up to 200 m.a.s.l. (Cisneros‐Heredia, 2006; Frost, 2023; Ron et al., 2004). The records presented here constitute the easternmost known record of the species, with the nearest locality being at a distance of 56 km (Santa Ana, Manabí Province; Pontificia Universidad Católica del Ecuador 2021a) (Figure 3c).

### Class amphibia: Order Anura: Family Strabomantidae

### *Pristimantis kuri* Yánez‐Muñoz, Sánchez‐Nivicela, and Reyes‐Puig, 2016

**New Records**. ECUADOR—**Bolivar** • Chillanes Canton, El progreso farm; 2°7′46.2″ S, 79°6′51.5″ W; alt. 918 m; 09.III.2023; Natalia Zapata‐Salvatierra leg; found perched on a trunk 180 cm high on a tourist trail to a waterfall; SVL 44 mm; 1 (sex indet.); ZSFQ5264. • Chillanes Canton, El Progreso farm; 2°7′45.1″ S, 79°6′52.6″ W; alt. 927 m; 09.III.2023; Keyko Cruz‐García leg; found perched on a branch 102 cm high on a tourist trail to a waterfall; SVL 42 mm; 1 (sex indet.); ZSFQ5265. **Cañar** • Cañar Canton, San Pablo town; 2°20′16.1″ S, 79°10′38.3″ W; alt. 463 m; 12.III.2023; Natalia Zapata‐Salvatierra leg; found perched on a branch at a height of 230 cm in a stream with secondary forest on both sides; SVL 23 mm; 1 (sex indet.); ZSFQ5188. • Cañar Canton, San Antonio de Paguancay parish, Ocaña; 2°29′23.6″ S, 79°11′5.6″ W; alt. 961 m; 29.VIII.2014; Juan C. Sánchez‐Nivicela, Verónica L. Urgiles leg; found perched among medium vegetation (not higher than 1.7 m) within a forest remnant; SVL 40.33 mm; 40.75 mm; 29.45 mm; 3 ♀; MZUA‐An.1319, MZUA‐An.1323, MZUA‐An.1329; SVL 29.25 mm; 1 ♂; MZUA‐An.1317; SVL 18.79 mm; 14.62 mm; two (juveniles); MZUA‐An.1320 and MZUA‐An.1321. • Cañar Canton, San Antonio de Paguancay parish, Ocaña; 2°29′3.7″ S, 79°15′12.2″ W; alt. 946 m; 29.VI.2016; Juan C. Sánchez‐Nivicela, Verónica L. Urgiles, Karla Neira, Amanda Quezada leg; found perched among medium vegetation (not higher than 1.7 m) within a forest remnant; SVL 29.62 mm; 1 ♀; MZUA‐An.1717; SVL: 15.87 mm; 17.93 mm; 10.91 mm; 3 (juveniles); MZUA‐An.1682, MZUA‐An.1718, and MZUA‐An.1721. – **Guayas** • El Triunfo Canton, Rancho Alemán; 2°20′13.9″ S, 79°12′44.3″ W; alt. 292 m; 08.III.2023; Nadia Chauca leg; found perched among trunk moss 150 cm above the ground in mountains with secondary forest; SVL 36.5 mm; 1 (sex indet.); ZSFQ5263.

**FIGURE 4:**
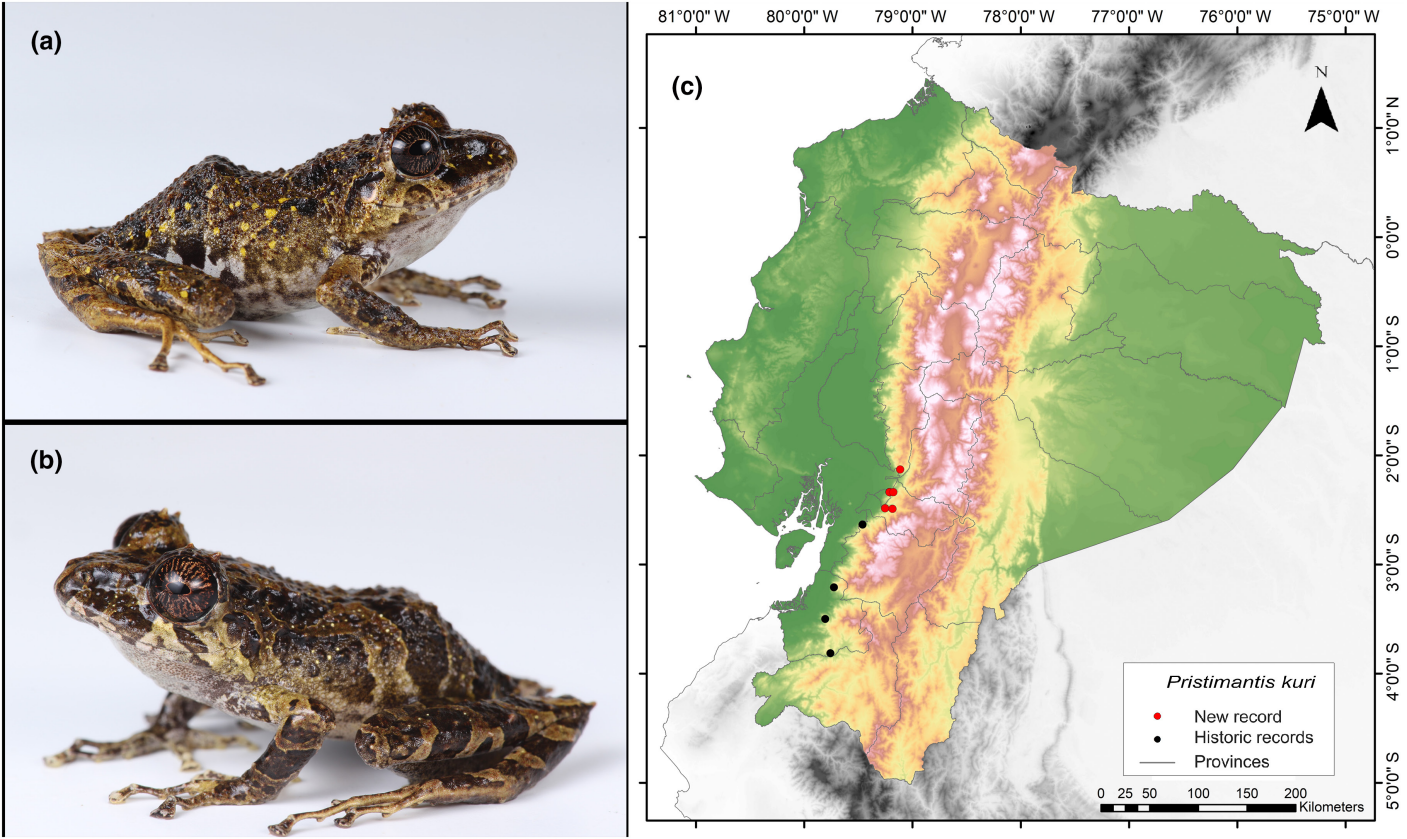
Photographs of *Pristimantis kuri* (a and b) and its distribution map (c) depicting historic records in black dots and the new record in red dots. The images belong to the specimen registered in this study.

**Identification.** (Figure 4a,b) It is a small frog; its back is variable between beige and brown and it presents a dermal crest in the scapular region in the shape of an “H”. It has a conical tubercle on the eyelid and heel, and several subconical ones on the tarsus, its toe discs are expanded, and it lacks a basement membrane between the toes. It has an average SVL range of 26.1 mm. The head presents an interorbital, canthal, and labial bars. The rear of the flanks, the groins, and the anterior surfaces of the legs are black or dark brown with white reticulations. The belly is variable between black and gray with solid white spots. The coloration of the iris is copper with black diagonal lines and a reddish horizontal midline (Yánez‐Muñoz et al., 2016).

**Distribution.** When initially described by Yánez‐Muñoz et al. (2016), this species was known only in two localities within forests in the southern region of the Western Cordillera of the Andes, specifically in the Catamayo‐Alamor area of the province of El Oro. Its altitudinal range extends 800–900 m.a.s.l. (Páez‐Rosales & Ron, 2022; Yánez‐Muñoz et al., 2016). According to the compilation of bibliography and database information, this species would be present in four locations. Our observation marks the northernmost occurrence of *P*. *kuri*, located 68 km north of the nearest recorded location for this species (Rio Tamarindo, Azuay Province; Pontificia Universidad Católica del Ecuador 2021a) (Figure 4c). This record also sets an altitudinal record for the species, as it was found at an altitude of 292 m.a.s.l., whereas the species has previously been reported at altitudes greater than 800 m.a.s.l.

### *Pristimantis muricatus* (Lynch and Miyata, 1980)

**New Records**. ECUADOR—**Cañar** • Cañar Canton, Cutuguay town; 2°18′27.39″ S, 79°7′54.73″ W; alt. 750 m; 06.II.2024; Natalia Zapata‐Salvatierra leg; found perched on a broken branch that was hanging from a tree full of moss 200 cm high and 150 cm from a stream; SVL 25.32 mm, 1 (sex indet.); MUTPL‐A 3652.

**FIGURE 5:**
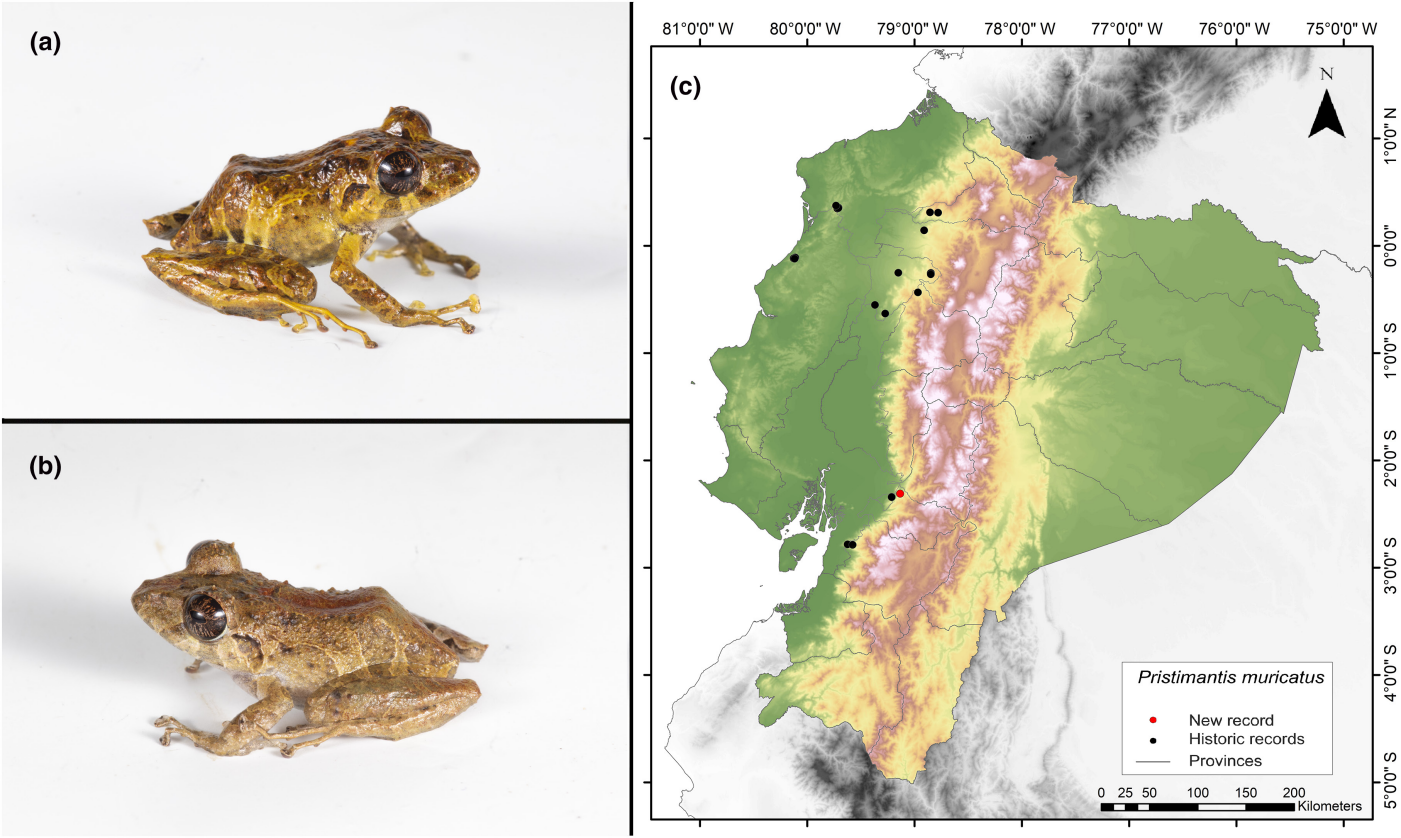
Photographs of *Pristimantis muricatus* (a and b) and its distribution map (c) depicting historic records in black dots and the new record in red dots. The images belong to the specimen registered in this study.

**Identification.** (Figure 5a,b) Males with an LRC between 31.8 and 40.7 mm; females between 36.8 and 46.3 mm. Snout subacuminate in dorsal view, rounded in profile. It is characterized by having smooth dorsal skin with many conical tubercles, including several enlarged and pointed tubercles toward the flanks, and external edges of the tarsus, heel, and upper eyelid. It has a moderately distinctive tympanic membrane and tympanic ring. The iris is golden‐copper with black radial stripes. Males usually present slits and vocal sacs; nuptial pads are absent. Finger I of the hand is shorter than Finger II, and has wide discs; fingers have lateral keels. Dorsal coloration is mostly dark brown, brown, reddish, or in shades of dark green with irregular yellow, light brown, or dark brown stripes toward the groin; may have a cream dorsal stripe. Flanks are pale yellowish‐brown with reddish hues toward the groin. The belly and ventral surfaces of legs have dark spots on a white background; some individuals may have yellowish spots (Frenkel, Varela‐Jaramillo, et al., 2022; Lynch & Miyata, 1980; MECN, 2010).

**Distribution.** It is distributed in the western foothills of the Andes mountain range in Ecuador. It is known from a few localities in the provinces of Cotopaxi, Esmeraldas, Guayas, Imbabura, Los Ríos, Manabí, Pichincha, and Santo Domingo. It has an altitudinal range from 220 to 1380 m.a.s.l. (Frenkel, Varela‐Jaramillo, et al., 2022; Frost, 2023; Instituto Nacional de Biodiversidad, 2022; MECN, 2010). The record presented here is the first documented record for the province of Cañar (Figure 5c).

### *Pristimantis nyctophylax* (Lynch, 1976)

**New Records**. ECUADOR—**Guayas** • El Triunfo Canton, Rancho Alemán; 2°20′13.6″ S, 79°12′46.8″ W; alt. 286 m; 06.X.2022; Keyko Cruz‐García leg; found in amplexus perched on a bush leaf over the water of a ravine at a height of 120 cm; SVL 33 mm, 22 mm; 1 ♂, 1 ♀; QCAZA78111, QCAZA78112. **Cañar** • Cañar Canton, El bosque del Amigo farm; 2°16′57.7″ S, 79°6′2.5″ W, alt. 783 m; 11.III.2023; Keyko Cruz‐García leg; found perched on a leaf petiole, 190 cm above the ground, over a temporary stream with secondary vegetation on the margins, and a significantly steep slope; SVL 17 mm; 1 (sex indet.); ZSFQ5267. • Cañar Canton, San Antonio de Paguancay parish, Ocaña; 2°29′23.8″ S, 79°11′5.8″ W; alt. 961 m; 25‐26.VIII.2014; Juan C. Sánchez‐Nivicela, Verónica L. Urgiles leg; found perched on leaves and small branches, between 60 cm and 150 cm, inside forest remnants; SVL 29.35 mm; 21.83 mm; 21.75 mm; 23.96 mm; 22.55 mm; 23.35 mm; and 24.83 mm; 7 ♂; MZUA‐An.1318, MZUA‐An.1324, MZUA‐An.1325, MZUA‐An.1326, MZUA‐An.1328, MZUA‐An.1330, and MZUA‐An.1393. • Cañar Canton, San Antonio de Paguancay parish, Ocaña; 2°29′3.7″ S, 79°15′12.2″ W; alt. 946 m; 28–29.VI.2016; Juan C. Sánchez‐Nivicela, Verónica L. Urgiles, Karla Neira, Amanda Quezada leg; found among shrubby vegetation, between 120 cm and 200 cm, within a remnant of forest; SVL 37.14 mm; 34.19 mm; 2 ♀; MZUA‐An.1681 and MZUA‐An.1720.

**FIGURE 6:**
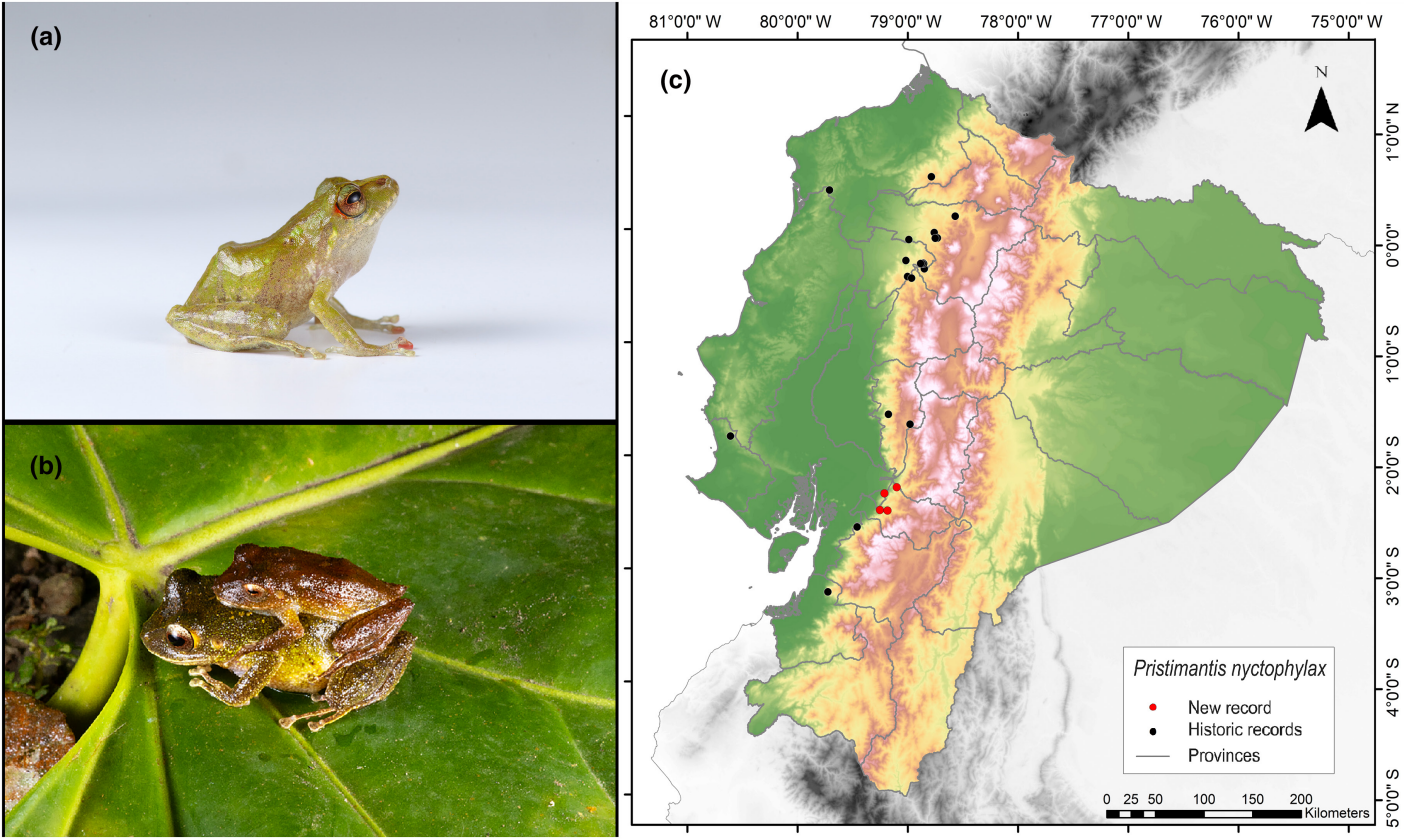
Photographs of *Pristimantis nyctophylax* (a and b) and its distribution map (c) depicting historic records in black dots and the new record in red dots. The images belong to the specimen registered in this study.

**Identification.** (Figure 6a,b) It is a small‐ to medium‐sized frog with a cream‐brown to brown back, expanded discs on the fingers, yellow or red sclera, and irises with well‐defined black reticulations. The SVL range is from 26.8 to 33.8 mm. It has slightly granular dorsal skin; belly ringed; prominent discoid fold; dorsolateral folds absent; prominent tympanic membrane and ring; and eardrum taller than long (Frenkel, Guayasamín, & Varela‐Jaramillo, 2022; Lynch & Duellman, 1997).

**Distribution.** It is distributed only on the western flanks of the Andes of Ecuador. It has been recorded in the provinces of Cotopaxi, Pichincha, and Santo Domingo de los Tsáchilas and less frequently in Azuay, Bolívar, Esmeraldas, Imbabura, and El Oro. The known altitudinal range is from 1140 to 2100 m.a.s.l. (Lynch & Duellman, 1997; Pontificia Universidad Católica del Ecuador 2021a; Yánez‐Muñoz et al., 2019). The present records address an information gap spanning 136 km, with the nearest northern locality to our observations situated 65 km away (Bosque Protector Cashca Totoras, Bolívar Province; Pontificia Universidad Católica del Ecuador 2021a) and the closest southern locality positioned at a distance of 52 km (San Miguel, Guayas Province; Instituto Nacional de Biodiversidad, 2022) (Figure 6c).

### *Pristimantis walkeri* (Lynch, 1974)

**New Records**. ECUADOR—**Cañar** • Cañar Canton, San Pablo town; 2°21′17″ S, 79°10′47.7″ W, alt. 519 m; 11.IX.2023; Keyko Cruz‐García; found perched on a bush leaf 150 cm high in the middle of the road covered with low vegetation; SVL 18.32 mm; 1 (sex indet.); MUTPL‐A 1659. • Cañar Canton, El bosque del Amigo farm; 2°17′7.8″ S, 79°6′19.8″ W, alt. 877 m; 11.III.2023; Nadia Chauca; found vocalizing on a branch at 100 cm height over a temporary intermittent stream with secondary vegetation on the margins and a significantly steep slope; SVL 17 mm; 1 (sex indet.); ZSFQ5212. • Cañar Canton, San Pablo town; 2°21′4.8″ S, 79°10′57.1″ W, alt. 636 m; 12.IX.2023; Natalia Zapata‐Salvatierra; found perched on a bush leaf 200 cm high on the side of a steep slope, it was vocalizing; SVL 18.29 mm; 1 ♂; MUTPL‐A 1654. • Cañar Canton, San Pablo town; 2°21′13.5″ S, 79°10′49.6″ W, alt. 610 m; 12.IX.2023; Natalia Zapata‐Salvatierra; found perched on a bush leaf in secondary mainland forest at 170 cm high; SVL 18.59 mm; 1 (sex indet.); MUTPL‐A 1661. • Cañar Canton, San Antonio de Paguancay parish, Ocaña; 2°29′57.01″ S, 79°14′52.35″ W; alt. 494 m; 23.IX.2015; Juan C. Sánchez‐Nivicela, Verónica L. Urgiles, Karla Neira, leg; found among low bushes between 70 cm and 160 cm, inside the forest; SVL 26.73 mm; 31.81 mm; SVL 20.33 mm; 27.82 mm; 19.05 mm; 5 ♀; MZUA‐An.3180, MZUA‐An.3181, MZUA‐An.3184, MZUA‐An.3193, MZUA‐An.3194; SVL 19.6 mm; 1 ♂; MZUA‐An.3186.

**FIGURE 7:**
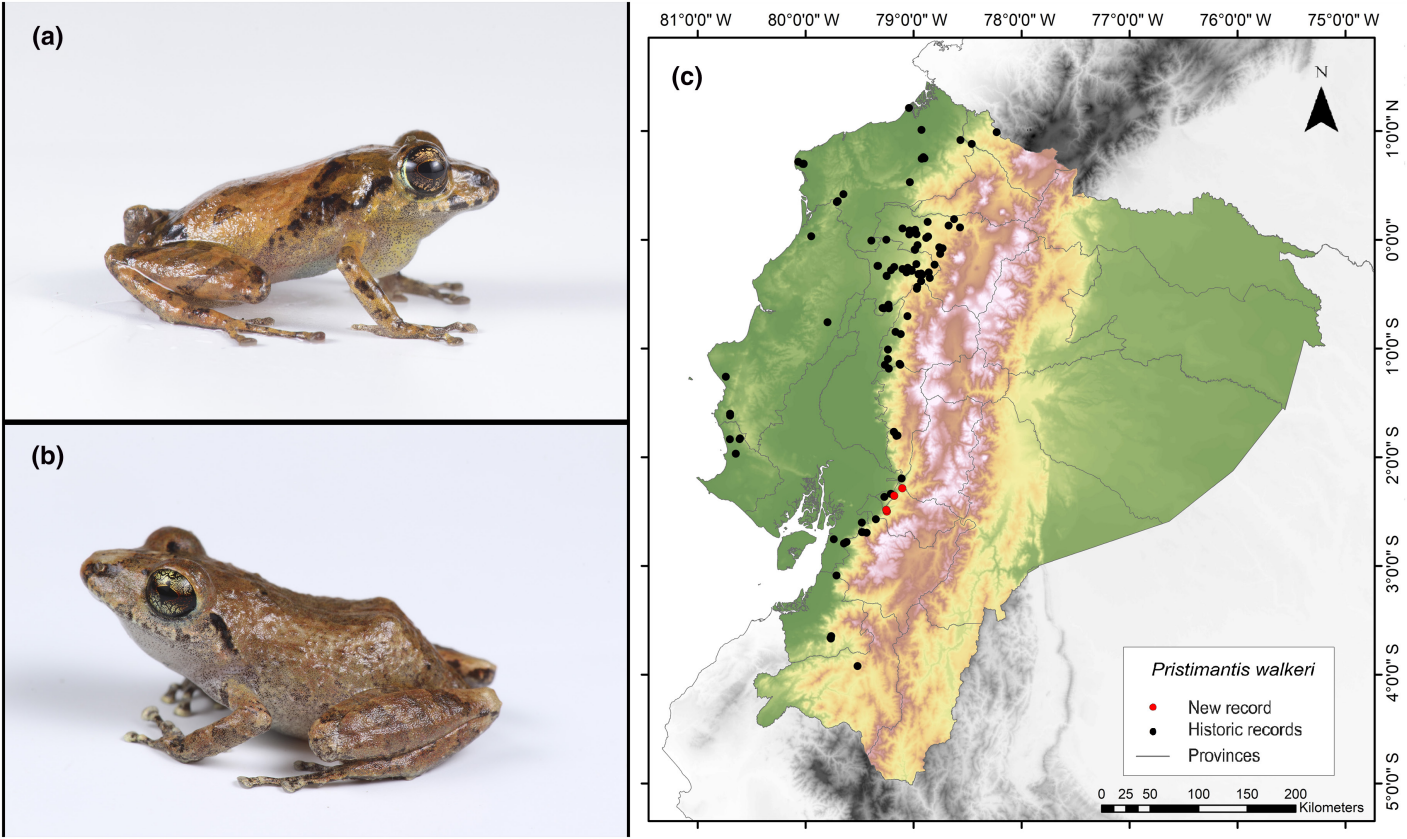
Photographs of *Pristimantis walkeri* (a and b) and its distribution map (c) depicting historic records in black dots and the new record in red dots. The images belong to the specimen registered in this study.

**Identification.** (Figure 7a,b) It is a very small frog with brown and groin coloration, and the hidden surfaces of the thighs have yellow‐to‐orange spots on a brown‐to‐gray background. The SVL range is from 16.2 to 21.6 mm. The discs of its fingers are widely expanded, lacking a basal webbing between the pedal digits. The dorsal skin is slightly granular, while the ventral surface displays an areolate pattern. There are no dorsolateral folds present. The tympanic membrane and annulus are prominent. The upper eyelid lacks tubercles. Finger I of the hand is shorter than Finger II. Weak tubercles are present on the tarsus. The fingers of the hands have cutaneous ridges. Toe V is much longer than Toe III (Lynch & Duellman, 1997).

**Distribution.** This species of frog is endemic to the western region of Ecuador and is found in the lowlands of Chocó and adjacent western foothills of the Ecuadorian Andes, suggesting a possible presence in Colombia. In Ecuador, it has been recorded in several provinces, including Azuay, Bolívar, Carchi, Cotopaxi, El Oro, Esmeraldas, Guayas, Loja, Los Ríos, Manabí, Pichincha, Santa Elena, and Santo Domingo de los Tsáchilas (Frost, 2023; Lynch, 1974; Pontificia Universidad Católica del Ecuador 2021a). The record presented here is the first documented record for the province of Cañar (Figure 7c).

### Class Reptilia: Order Squamata: Family Sphaerodactylidae

### *Lepidoblepharis buchwaldi* Werner, 1910

**New Records**. ECUADOR—**Cañar** • Cañar Canton, El bosque del Amigo farm; 2°16′42.6″ S, 79°6′42.1″ W; alt. 810 m; 11.III.2023; Keyko Cruz‐García leg; found foraging in leaf litter beside the road; SVL 26.3 mm; 1 ♂; ZSFQ5272. **El Oro** • El Guabo Canton, Las Cascadas de Manuel; 3°12′22.4″ S, 79°44′12.2″ W; alt. 182 m; 07.VI.2014; Juan Carlos Sánchez, Verónica L. Urgiles leg; found among the fallen leaves on the ground, near one of the waterfalls; SVL 23.87 mm; 1 ♂; MZUA‐Re.0213.

**FIGURE 8:**
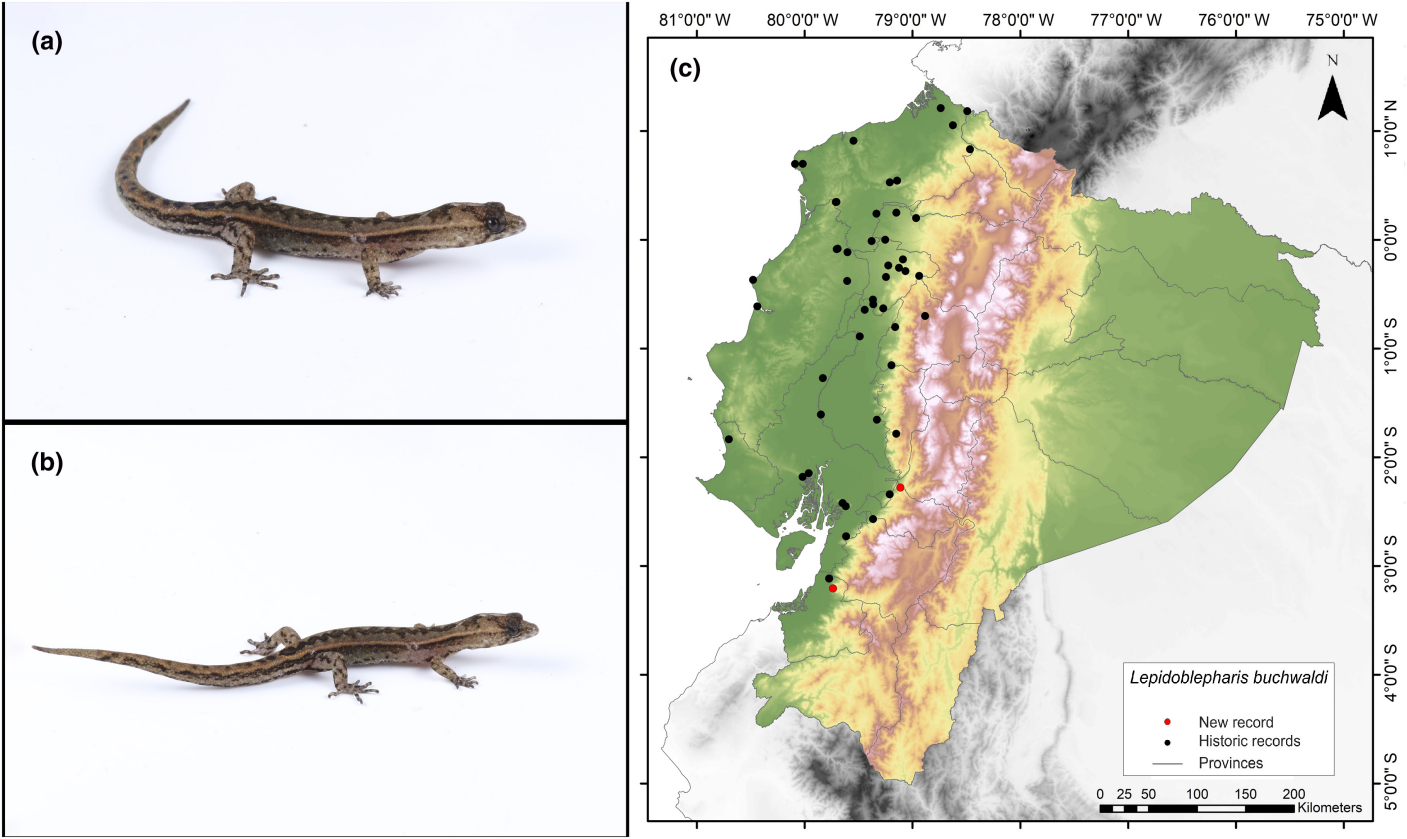
Photographs of *Lepidoblepharis buchwaldi* (a and b) and its distribution map (c) depicting historic records in black dots and the new record in red dots. The images belong to the specimen registered in this study.

**Identification.** (Figure 8a,b) It is a small‐sized lizard with homogeneous juxtaposed dorsal scales, which are small but larger than the scales on the surface of the head; these scales can be smooth or with a slightly pronounced keel. The lamellae on the fourth toe range from 9 to 11. The mental scale has a concave posterior edge with the margin forming an inverted V shape, with two notches present or absent (Calderón‐Espinosa & Medina‐Rangel, 2016; Peters & Donoso‐Barros, 1970).

**Distribution.** This species is endemic to an estimated area of 46,529 km^2^ in the Tumbesian lowlands and adjacent foothills of the Andes in Ecuador, at altitudes ranging from 4 to 1029 m.a.s.l. In Ecuador, it has been reported in the provinces of El Oro, Guayas, Los Ríos, Cotopaxi, Manabí, Esmeraldas, Santo Domingo de los Tsáchilas, and Azuay (Arteaga, 2021; Ávila‐Pires, 2001; Peters & Donoso‐Barros, 1970; Torres‐Carvajal, 2001; Yánez‐Muñoz et al., 2019). This record is the first documented record of this species in the Cañar Province (Figure 8c).

### Class Reptilia: Order Squamata: Family Colubridae

### *Chironius flavopictus* (Werner, 1909)

**New Records**. ECUADOR—**Guayas** • El Triunfo Canton, Finca La Meca Tabamesa; 2°16′42.4″ S, 79°13′4.6″ W; alt. 128 m; 26.VI.2023; Keyko Cruz‐García leg; found asleep perched among guadúa cane branches about 5 m high above a water stream with grasslands on both banks; SVL 1200 mm; 1 ♀; ZSFQ6368. **Cañar** • Cañar Canton, La Troncal; 2°24′52.32″ S, 79°20′51.68″ W; alt. 68 m; 23.XII.2019; Juan C. Sánchez‐Nivicela, Lorena Orellana, Juan M. Orellana, obs.; found near a stream on a road.

**FIGURE 9:**
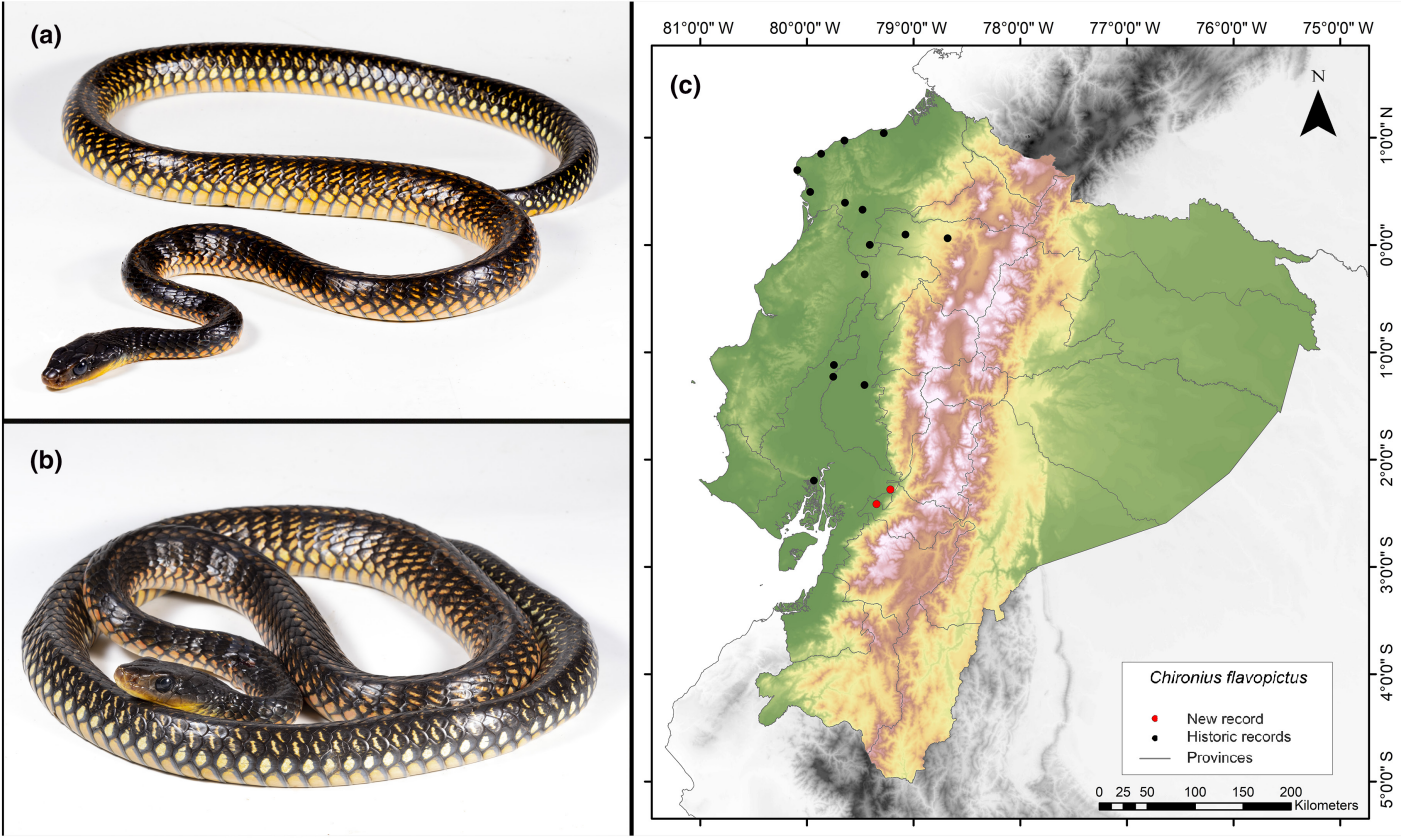
Photographs of *Chironius flavopictus* (a and b) and its distribution map (c) depicting historic records in black dots and the new record in red dots. The images belong to the specimen registered in this study.

**Identification.** (Figure 9a,b) This species is characterized by having 12 rows of dorsomedial scales; divided anal plate; relatively consistent reduction of dorsal scale rows 12‐12‐8; a white or yellow dot or spot on most dorsal scales (Dixon et al., 1993).

**Distribution.** This species is distributed in evergreen rainforests along the Pacific Coast of Ecuador, Colombia, Panamá, and southern provinces of Costa Rica. In Ecuador, this species has been reported in the provinces of Esmeraldas, Guayas, Los Ríos, Manabí, Pichincha, and Santo Domingo de los Tsáchilas, from 0 to 566 m.a.s.l. (Dixon et al., 1993; Rodríguez‐Guerra, 2020). This study presents the southernmost record of *C*. *flavopictus* to date, extending its known distribution 79 km further south than previously documented (Guayaquil, Guayas Province; Dixon et al., 1993) (Figure 9c). While the lectotype designated for this species originates from Guayaquil in the vicinity of the 1900s, the southernmost record since then is 111 km from our current observation (Recinto La Muralla, Los Ríos Province, Pontificia Universidad Católica del Ecuador 2021b). Therefore, we consider it important to report this record due to the limited information available for this species, which is classified as Data Deficient by the IUCN (2024).

### *Chironius grandisquamis* (Peters, 1869)

**New Records**. ECUADOR—**Guayas** • El Triunfo Canton, Rancho Alemán; 2°20′10.7″ S, 79°12′51.3″ W; alt. 272 m; 16.XII.2023; Natalia Zapata‐Salvatierra leg; found asleep perched on a branch of a medium‐sized bush at 150 cm next to a steep and muddy path due to the constant drizzle in the area; SVL 500, 9 mm; 1 ♂; MUTPL‐R 474. • Naranjal Canton, Cerro de Hayas; 2°44′12.5″ S, 79°37′39.9″ W; alt. 5 m; 29.XII.2015; Keyko Cruz‐García obs.; found foraging among rocky water ravine. **Cañar** • Cañar Canton, San Antonio de Paguancay parish, Ocaña; 2°29′56.7″ S, 79°14′52.4″ W; alt. 495 m; 24. IX.2015; Juan C. Sánchez‐Nivicela, Verónica L. Urgiles, Karla Neira obs.; found during the night among shrubby vegetation, at 250 cm, in the forest.

**FIGURE 10:**
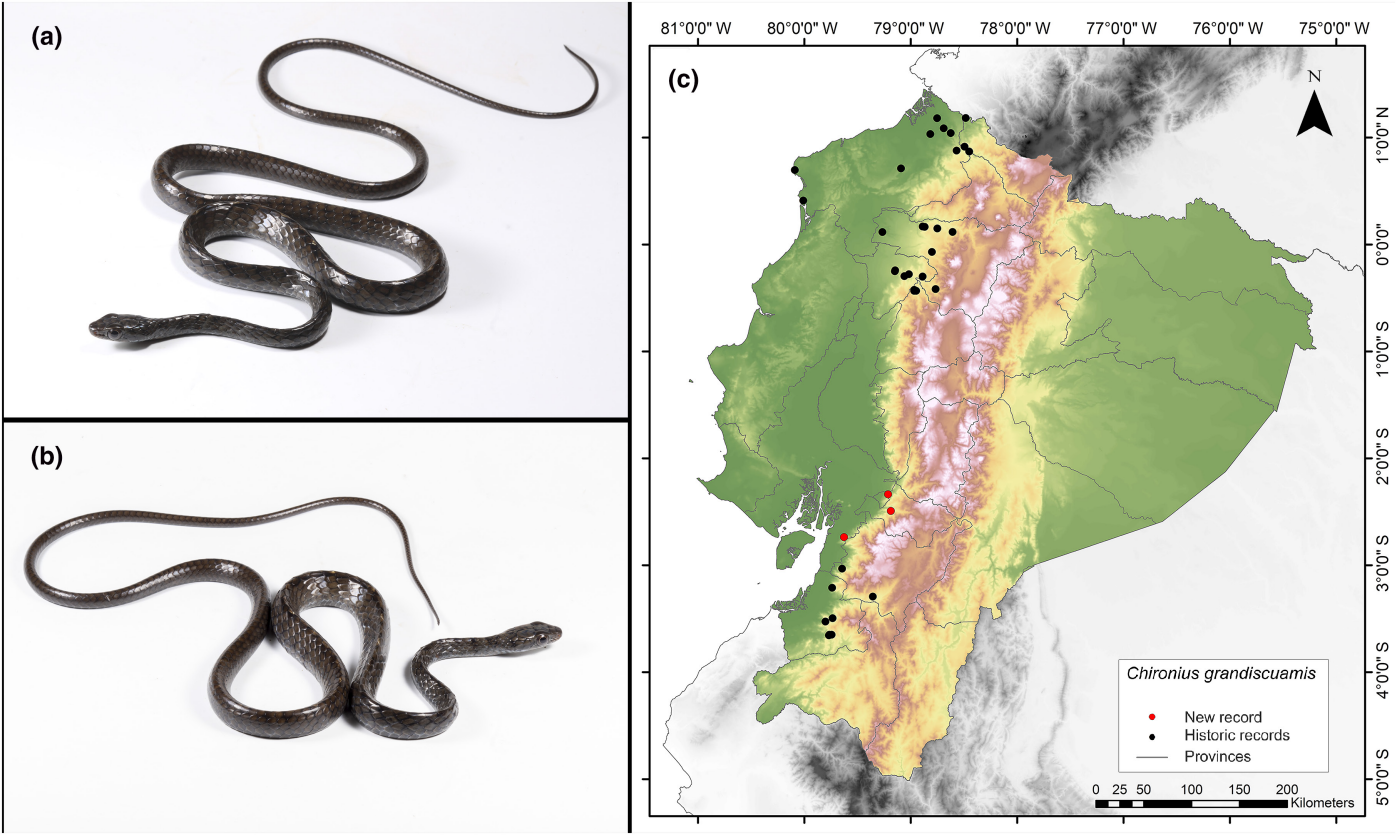
Photographs of *Chironius grandisquamis* (a and b) and its distribution map (c) depicting historic records in black dots and the new record in red dots. The images belong to the specimen registered in this study.

**Identification**. (Figure 10a,b) This species is recognized by its 10 rows of scales on its upper back, a divided anal plate, and a dorsal pattern that can exhibit either striping or be entirely black. In juveniles, the ventral surface is white, while in adults, the anterior portion of the ventrum is white, transitioning to black toward the posterior. The scales along the sides of the spine also have a ridge (Dixon et al., 1993). It is important to mention that the collected specimen (MUTPL‐R 474) is a subadult individual with a dark brownish color. In addition, it does not present keeled paraventral scales, nor do the individuals collected and shown in the database of Pontificia Universidad Católica del Ecuador (2021b) have this characteristic.

**Distribution.** This species is found from Honduras to Colombia's Magdalena Valley along the Atlantic versant, and from the Pacific slope of central Costa Rica through the Pacific lowlands of Colombia to Ecuador (Savage, 2002). In Ecuador, it has been reported in the provinces of Cotopaxi, El Oro, Esmeraldas, Imbabura, Santo Domingo de los Tsáchilas, Pichincha, Azuay y Manabí (Garzón‐Santomaro et al., 2019; Pontificia Universidad Católica del Ecuador 2021b). The present records address an information gap spanning 295 km, with the nearest northern locality to our record situated 212 km away (San Francisco de Las Pampas, Cotopaxi Province; Pontificia Universidad Católica del Ecuador 2021b), and the closest southern locality positioned at a distance of 36 km (Vía Camilo Ponce Enríquez‐Coca, Azuay Province; Pontificia Universidad Católica del Ecuador 2021b) (Figure 10c).

### *Dendrophidion graciliverpa* (Cadle, 2012)

**New Records**. ECUADOR—**Cañar** • Cañar Canton, El bosque del Amigo farm; 2°16′58.8″ S, 79°6′9.4″ W; alt. 831 m; 11.III.2023; Keyko Cruz‐García leg; found foraging in leaf litter near the edge of the road in the middle of the mountain.; SVL 285 mm; 1 (sex indet.); ZSFQ5274 • Cañar Canton, San Pablo Town; 2°20′8.6″ S, 79°10′53″ W; alt. 549 m; 13.IX.2023; Keyko Cruz‐García leg; foraging in leaf litter in secondary forest in the upper part of a steep mountain; SVL 503 mm; 1 ♀; MUTPL‐R 460.

**FIGURE 11:**
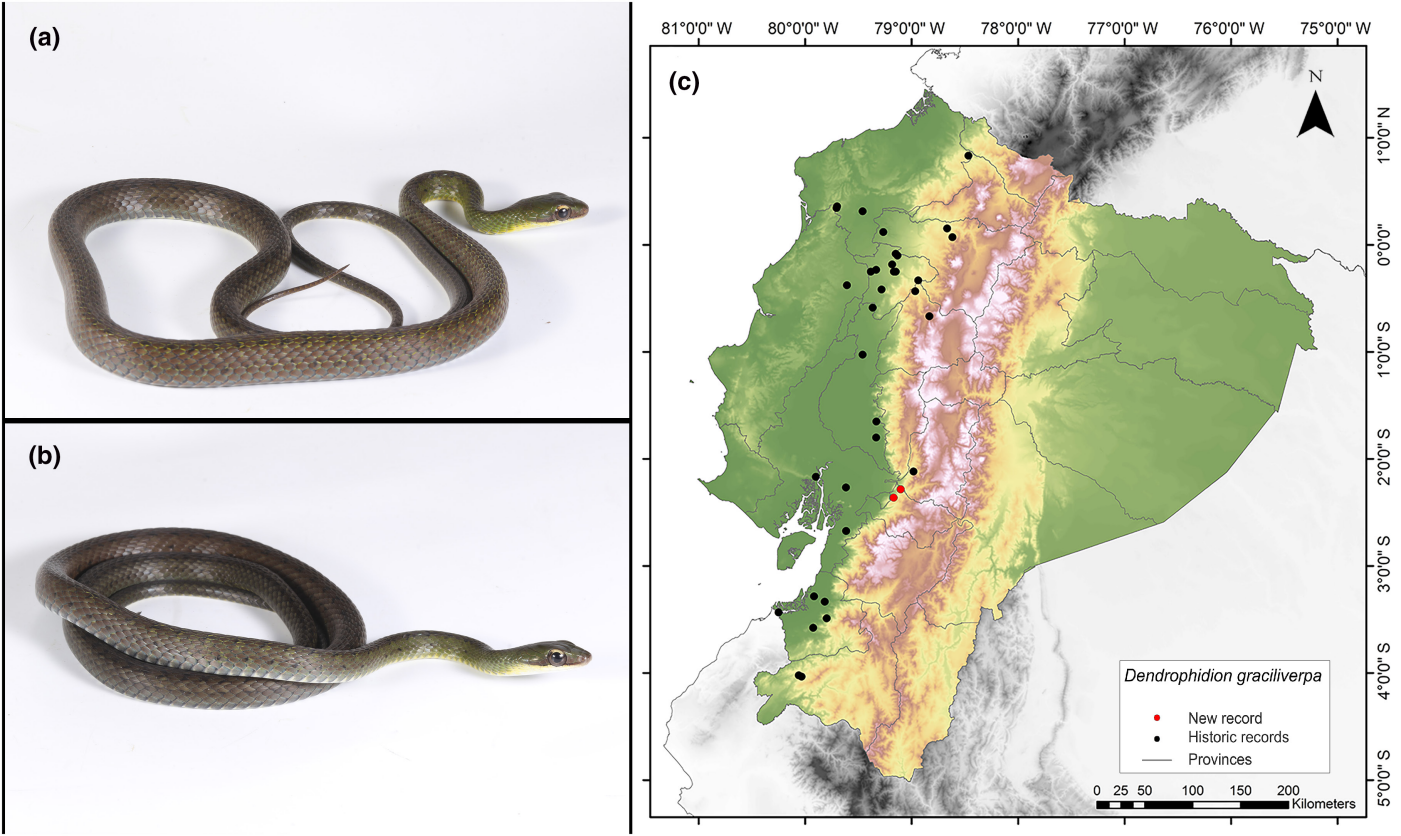
Photographs of *Dendrophidion graciliverpa* (a and b) and its distribution map (c) depicting historic records in black dots and the new record in red dots. The images belong to the specimen registered in this study.

**Identification.** (Figure 11a,b) This serpent displays a reduction in the number of scales along both the upper and lower surfaces of its tail, decreasing from 8 to 6 in the area before subcaudal scale 28 (with a range of 7–27). It possesses a divided anal plate, with a subcaudal count equal to or greater than 120 in both males and females. Subadults specimens often display narrow pale bands or clusters of eye‐like markings, while adults maintain these patterns or adopt a predominantly brown or green coloration (with more than 55 pale bands across the body). The ventral scales are either plain or feature slender dark lines along the front edge of each plate. In its living state, the snake has a greenish‐brown to green head and a body that is brown, olive, or grayish. The hemipenis, when everted, is characterized by an exceptionally long and slim proximal section and an expanded distal portion adorned with spines, calyces, and other apical decorations. The total number of enlarged spines on the hemipenis is 80 (Cadle, 2012). It is worth noting that the collected specimen (MUTPL‐R 460) exhibits black spots along the dorsum that fade as they move away from the individual's head. This characteristic is not textually described in Cadle (2012). However, in the photos shown in said article of the holotype of this species, you can see these patterns of black dots. Furthermore, the subject exhibited in the anterior region of its body and the anterior and/or posterior portions of the scales demonstrate marbled black or vibrant yellow edges with a more prominent brown center toward the posterior aspect of each scale; and the lower 12–14 dorsal rows on the neck lack keelsas depicted in Figure 11a,b.

**Distribution.** This snake is endemic to the lowland regions of western Ecuador, from sea level up to 1865 m.a.s.l. It has been documented in several provinces, including Azuay, Esmeraldas, Imbabura, Pichincha, Santo Domingo de los Tsáchilas, Cotopaxi, Chimborazo, Guayas, Manabí, Los Ríos, El Oro, and Loja (Cadle, 2012; Garzón‐Santomaro et al., 2019). The record presented here is the first documented record for the province of Cañar (Figure 11c).

### *Imantodes inornatus* (Boulenger, 1896)

**New Records**. ECUADOR—**Guayas** • El Triunfo Canton, Rancho Alemán; 2°20′18.27″ S, 79°12′46.85″ W; alt. 338 m; 15.XII.2023; Keyko Cruz‐García leg; found foraging between branches of a medium‐sized bush 206 cm above the ground, next to a muddy road due to the constant drizzle in the locality; SVL 528.21 mm; 1 ♀; MUTPL‐R 473.

**FIGURE 12:**
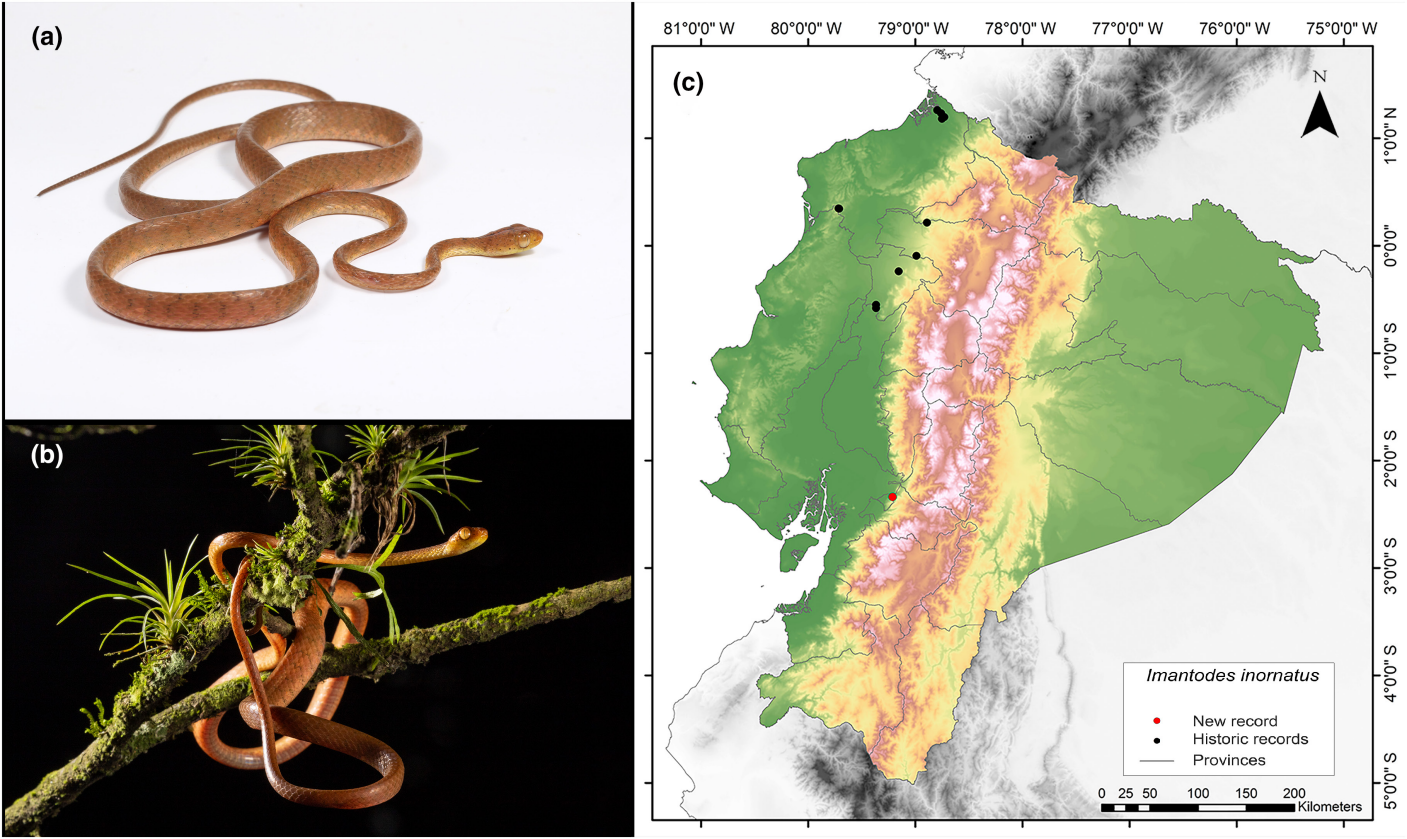
Photographs of *Imantodes inornatus* (a and b) and its distribution map (c) depicting historic records in black dots and the new record in red dots. The images belong to the specimen registered in this study.

**Identification.** (Figure 12a,b) This species is distinguished by its dorsal pattern, which typically consists of small dark spots and specks, occasionally forming very small transverse bars that do not extend beyond a single row of scales. Males have 199–218 ventral scales, while females have 196–212. Normally, there is a single preocular scale, although the occurrence of two is rare. Similarly, there are typically two postocular scales, although three are rare. The supralabials range from 7 to 9, usually settling at 8, with two or three adjacent to the orbit. Infralabials vary between 8 and 11, usually around 10. The number of temporals is variable (3–6 scales per side), typically appearing as 2 + 3. The usual count for dorsal scale rows is 17‐17‐17, although females may exhibit 17‐19‐17 or males 17‐17‐15 or 17‐17‐13. The vertebral scales are moderately enlarged, measuring 1.2–1.5 times the size of the medial–lateral scales. The anal plate is typically divided, although occasionally it may be entire (MECN, 2010; Pazmiño‐Otamendi, 2020a; Savage, 2002).

**Distribution.** *I*. *inornatus* is distributed in the lowlands and adjacent premontane foothills on the Caribbean slope from northwestern Honduras to eastern Panama; on the Pacific slope southwest of Costa Rica and southwest of Panama; and from the eastern end of Panama toward Ecuador. It lives in the tropical and western subtropical zones in an altitudinal range 5–1450 m.a.s.l. In Ecuador, it has been reported in the provinces of Esmeraldas, Los Rios, Manabi, Pichincha, and Santo Domingo de los Tsáchilas; and its altitudinal range does not exceed 800 m.a.s.l. (MECN, 2010; Pontificia Universidad Católica del Ecuador 2021b; Savage, 2002). Our record represents the northernmost record of *I*. *inornatus*, situated 196 km south of the closest record for this species (Centro Científico Río Palenque, Los Ríos Province; GBIF, 2024) (Figure 12c).

### *Ninia schmidti* (Arteaga and Harris, 2023)

**New Records**. ECUADOR—**Guayas** • Naranjal Canton, Cerro de Hayas; 2°44′12.5″ S, 79°37′44.4″ W; alt. 139 m; 12.VI.2016; Keyko Cruz‐García leg; found foraging on rocky terrain alongside a stream; SVL 485 mm, CL 83 mm; 1 ♂; MZUA.RE 0380.

**FIGURE 13:**
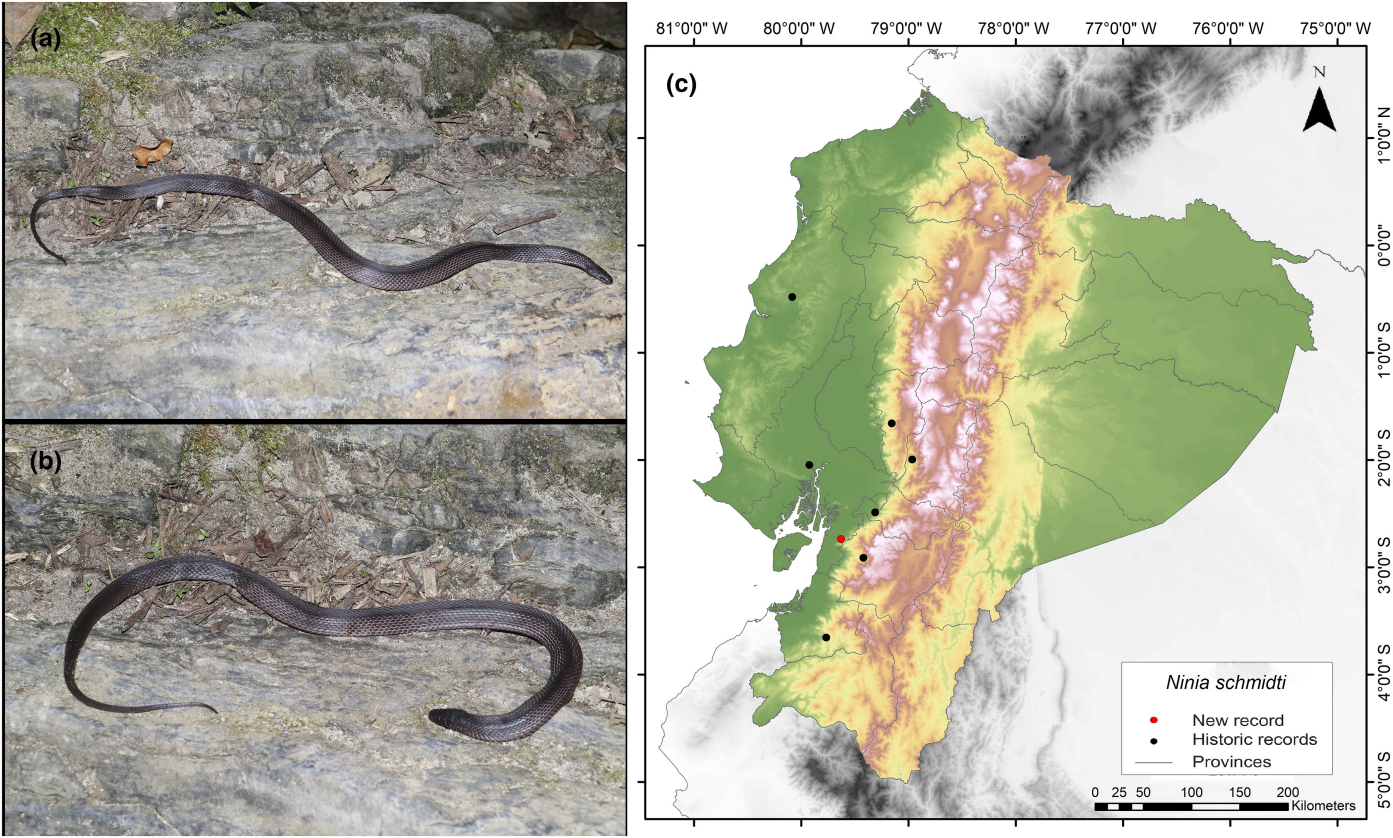
Photographs of *Ninia schmidti* (a and b) and its distribution map (c) depicting historic records in black dots and the new record in red dots. The images belong to the specimen registered in this study.

**Identification.** (Figure 13a,b) This species is characterized by the following combination of characters: 19/19/19 keeled dorsals; temporals 1 + 2; seven supralabials, with the third and fourth contacting the orbit; two postoculars; loreal scale 1.4–2.3 times longer than high; seven or eight infralabials, with the first four or five contacting chin shields; typically two rows of chin shields; one or two preventrals; 138–144 ventrals in males, 139–155 in females; 50–57 subcaudals in males, 46–53 in females; males measure 167–283 mm in snout–vent length (SVL), while females measure 230–409 mm; males have a head length (CL) of 42–61 mm, and females have a head length of 53–84 mm; uniform black dorsal coloration without a white nuchal collar; ventral surfaces of adults obscured by dark pigment, particularly along the posterior edge of each ventral scale, while some juveniles display immaculate white ventral surfaces (Arteaga & Harris, 2023).

**Distribution.** *N*. *schmidti* is endemic to an estimated area of 42,281 km^2^ along the Chocoan–Tumbesian transition area in western Ecuador. This species is only known from seven records within the provinces of Azuay, Bolivar, Chimborazo, El Oro, Guayas, and Manabí. In addition, it has been recorded at elevations from 46 to 1843 m.a.s.l. (Arteaga & Harris, 2023) (Figure 13c). The holotype designated for this species originates from Guayaquil around the year 1862. However, according to Arteaga and Harris (2023), this specimen was destroyed. Therefore, they assigned a new neotype from the same locality of Guayaquil, collected between 1901 and 1902. These two collected records were the only ones made in the Guayas Province. The neotype is located 84 km away from our current record. Hence, we consider it significant to report this discovery due to the limited available information concerning this newly described species.

### *Urotheca fulviceps* (Cope, 1886)

**New Records**. ECUADOR—**Guayas** • Naranjal Canton, Cerro de Hayas; 2°43′36.5″ S, 79°37′32.9″ W; alt. 242 m; 01.IX.2022; Keyko Cruz‐García leg; found foraging in the litter of secondary forest intervened by livestock; SVL 243 mm; 1 ♂; ZSFQ5546. **Cañar** • Cañar Canton, San Pablo town; 2°21′4.8″ S, 79°10′58.2″ W; alt. 565 m; 11.IX.2023; Nadia Chauca leg; Foraging in leaf litter in secondary forest very close to a steep slope; SVL 226 mm; 1 ♀; MUTPL‐R 462.

**FIGURE 14:**
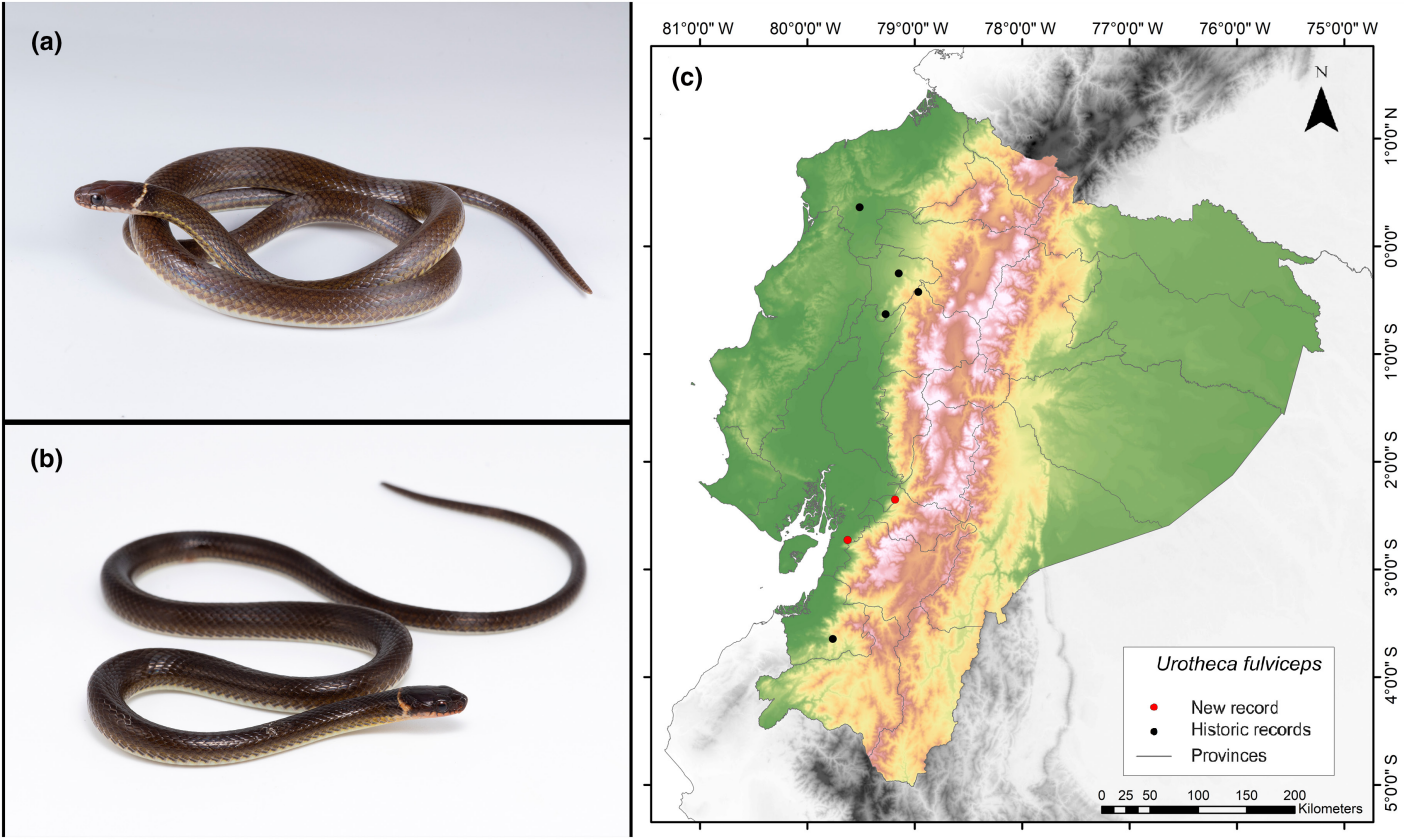
Photographs of *Urotheca fulviceps* (a and b) and its distribution map (c) depicting historic records in black dots and the new record in red dots. The images belong to the specimen registered in this study.

**Identification.** (Figure 14a,b) It is a small and slender snake with a cylindrical body, characterized by smooth scales arranged in 17 rows. The midbody and tail are relatively long, comprising 39%–45% of the total length. The back is usually uniform brown, occasionally featuring a pair of thin pale stripes or a row of white “dashed” markings along the first scale row, while the belly is immaculately white. The iris is reddish brown, with a golden tinge above, and a black tongue with yellowish‐gray tips (Leenders, 2019; MECN, 2010; Pazmiño‐Otamendi, 2020b; Savage, 2002; Toro‐Cardona, 2021). The individual in Figure 13a exhibited a brown coloration on the head with a prominent white nuchal collar and a cream–brown coloration on the infralabial scales. Additionally, it displayed a brownish coloration on the dorsum with a slight yellowish line above the paraventral scales. In contrast, the individual in Figure 13b showed a darker brown coloration on its head, with infralabial scales of a subtle orange tint and a cream‐yellowish nuchal collar. Furthermore, the dorsum of this individual appeared dark brown, with a faint longitudinal line observed above the paraventral scales.

**Distribution.** This species is distributed throughout the Mesoamerican lowlands of Central America and the Chocó Valley and Río Magdalena regions of northern South America. In Ecuador, it has been reported in the provinces of El Oro, Esmeraldas, Pichincha, and Cotopaxi. The altitudinal range of this species covers from approximately 0 to 1498 m.a.s.l. (González‐Maya et al., 2011; Toro‐Cardona, 2021; Wallach et al., 2014). The present records address an information gap spanning 341 km, with the nearest northern locality to our record situated 189 km away (Centinela, Santo Domingo de los Tsáchilas Province; GBIF, 2024), and the closest southern locality positioned at a distance of 101 km (Reserva Biológica Buenaventura, El Oro Province; Instituto Nacional de Biodiversidad, 2022) (Figure 14c).

## 4 DISCUSSION

In this manuscript, we present significant updates on the distribution of 14 species from western Ecuador, comprising seven amphibians and seven reptiles. These findings contribute substantially to our understanding of species with limited documentation by providing new localities for *Agalychnis spurelli*, *Chironius grandisquamis*, *Dendrophidion graciliverpa*, *Engystomops montubio*, *Hyloscirtus alytolylax*, *Lepidoblepharis buchwaldi*, *Pristimantis kuri*, *Pristimantis muricatus*, *Pristimantis nyctophylax*, *Pristimantis walkeri*, and *Urotheca fulviceps*. These updates help fill crucial information gaps regarding the distribution of these species and represent the first documented reports for the provinces of Bolívar, Guayas, and Cañar. Additionally, we provide a report on *Chironius flavopictus* and *Ninia schmidti*, both of which are species that have been poorly documented.

We present some interesting cases, such as the one involving *N*. *schmidti*, where a total length of 493 mm was previously documented according to Arteaga and Harris (2023). Nonetheless, our documented individual measured a substantial 568 mm in total length (SVL = 485 mm; CL = 83 mm), making it the largest individual reported to date, constituting a notable addition to the knowledge of this species. It is important to note that, due to a recent update of the genus *Ninia*, we request the review of the samples of *N*. *schmidti* and *N*. *guytudori* at the Zoology Museum of the Universidad San Francisco de Quito (ZSFQ), as mentioned by Arteaga and Harris (2023), to accurately identify our specimens for this article. Surprisingly, we were informed that *N*. *guytudori* specimens (including the holotype) are not deposited within the ZSFQ as indicated in that article. Therefore, we maintain reservations about its accuracy. However, it is important to emphasize that the main focus of our article is the extension of the distribution area of this species and not to discuss taxonomic proposals.

Our findings include the distribution extension of *E*. *montubio* to the province of Los Ríos, *Pristimantis muricatus* recorded in the province of Cañar, the southernmost recorded occurrence of *L*. *buchwaldi* in the province of El Oro, and the northernmost documented record of *P*. *kuri* in the province of Bolivar. The significance of these findings is amplified by the inherent threats to these habitats due to agricultural expansion, logging, mining, and poaching (Griffis‐Kyle et al., 2018). With these ecosystems at risk, understanding the distribution patterns of species becomes imperative for effective conservation strategies. Additionally, the endangered status of *P*. *kuri* according to the IUCN (2024) and the Ecuadorian Red List of Amphibians (Ortega‐Andrade et al., 2021), combined with declining populations due to habitat degradation, accentuates the urgency of conservation efforts, given its status as an endemic species. Another species facing great pressure due to human activities is *Agalychnis spurrelli*. Our registry begins to fill an important information gap of ~330 km. Similarly, the species *Pristimantis muricatus*, which is in the Vulnerable category both in the IUCN (2024) and in the Red List of Amphibians of Ecuador (Ortega‐Andrade et al., 2021), since this species is not widely distributed in western Ecuador, documenting new populations is important for the conservation of this species.

On the other hand, *Urotheca fulviceps* was previously documented by G. Onore in the vicinity of the area we are reporting on. However, concerns have arisen regarding the accuracy of the data, particularly concerning the altitudinal range, as indicated by Cisneros‐Heredia and Touzet (2004). However, with the current record, the existence of this species throughout this entire area would be confirmed. The record presented in this article contributes significantly to filling a gap in the distribution of this species, expanding our understanding of its range by approximately 300 km, and confirms the record made by G. Onore for the province of Guayas in 1988.

One of our last records, on December 15, 2023, was *Imantodes inornatus*, a snake that had only been reported in northwestern Ecuador. With this discovery, more than 250 km away, we not only expanded the distribution but also provided a better understanding of the ecology of this species. In addition, this record contributes for an adequate evaluation of the status of conservation, since it is currently listed as Least Concern (LC) in the IUCN (2024) and Data Deficient (DD) in the Red Book of Reptiles of Ecuador (Carrillo et al., 2005).

Continued research and monitoring efforts play a crucial role in understanding the diverse herpetofauna communities within Ecuador's endangered ecosystems. The expansion of distribution ranges, the identification of novel occurrences, and the documentation of species in previously unexplored areas contribute significantly to more robust conservation strategies aimed at preserving biodiversity. The humid forests located in the western foothills of Ecuador, in particular, face imminent threats due to alarming demographic, agricultural, and livestock expansions. However, these forests hold significant biological value due to the abundance of species and notable levels of endemism, as highlighted by Parker and Carr (1992). Consequently, the findings presented in this study underscore the pressing need for scientific inventories in the region.

## AUTHOR CONTRIBUTIONS

**Keyko Cruz‐García:** Conceptualization (lead); data curation (lead); formal analysis (lead); funding acquisition (supporting); investigation (equal); methodology (equal); project administration (equal); resources (lead); software (lead); supervision (lead); validation (lead); visualization (lead); writing – original draft (lead); writing – review and editing (lead). **Natalia Zapata‐Salvatierra:** Data curation (equal); formal analysis (equal); funding acquisition (equal); investigation (equal); methodology (lead); writing – original draft (equal); writing – review and editing (equal). **Juan C. Sánchez‐Nivicela:** Conceptualization (lead); data curation (lead); formal analysis (equal); funding acquisition (equal); investigation (equal); methodology (equal); validation (equal); writing – original draft (equal); writing – review and editing (equal). **Nadia Chauca:** Conceptualization (supporting); formal analysis (supporting); investigation (supporting); methodology (supporting); writing – original draft (supporting); writing – review and editing (supporting). **Sascha Matecki:** Funding acquisition (equal); investigation (supporting); methodology (supporting); project administration (supporting); resources (equal); writing – original draft (equal); writing – review and editing (equal). **Julian Perez‐Correa:** Conceptualization (equal); funding acquisition (equal); project administration (supporting); validation (equal); writing – original draft (equal); writing – review and editing (equal).

## CONFLICT OF INTEREST STATEMENT

Authors declare no conflict of interest in this manuscript.

### OPEN RESEARCH BADGES

This article has earned Open Data and Open Materials badges. Data and materials are available at doi:10.5061/dryad.kh1893do.

## Data Availability

The data that support the findings of this study and the historical records of the species are openly available in the DRYAD repository at doi:10.5061/dryad.kh18932dw.
